# Water Accelerates in the Hydration Shell of the N- and C‑Terminal Domains of α‑Synuclein in the Presence of NaCl

**DOI:** 10.1021/acs.jpcb.5c06647

**Published:** 2026-01-21

**Authors:** Stephen J. Koehler, Valerie Vaissier Welborn

**Affiliations:** † Department of Chemistry, 1757 Virginia Tech, Blacksburg, Virginia 24061, United States; ‡ Macromolecules Innovation Institute, Virginia Tech, Blacksburg, Virginia 24061, United States

## Abstract

α-Synuclein (α-Syn) is an intrinsically disordered protein (IDP) whose aggregation into fibrils is implicated in Parkinson’s disease (PD). While benign α-Syn aggregation frequently occurs, off-target aggregates are implicated in disease progression. Although most mechanisms of toxic α-Syn aggregate formation are unknown, high concentrations of salt ions have been shown to systematically result in faster aggregation. Previous work suggests that salt slows water in the hydration shell of α-Syn, promoting intermolecular interactions. Here, we use polarizable molecular dynamics (MD) to investigate the interactions between α-Syn and water in response to an increased NaCl concentration. While we also find that the water in the hydration shell of the nonamyloid-β component (NAC) domain slows down with increasing salt concentration, the water in the hydration shell of the N- and C-terminal domains accelerates. The segments of the N- and C-terminal domains that show faster water diffusion kinetics corroborate with truncation experiment results. Overall, our work suggests that α-Syn aggregation is related to partial salt-induced dehydration of the N- and C-terminal domains.

## Graphical abstract

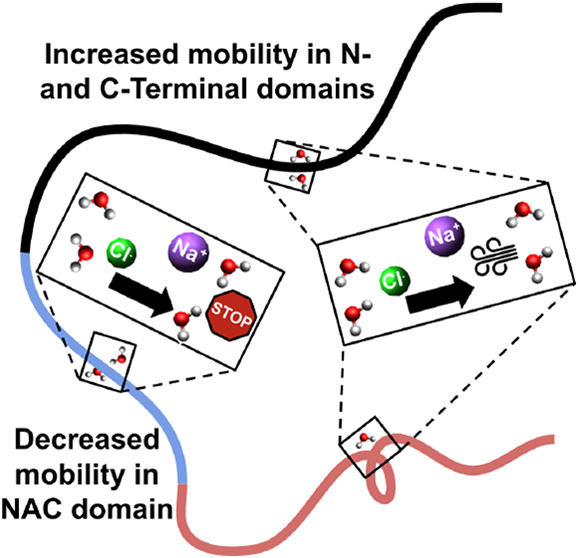

## Introduction

Human α-synuclein (α-Syn) is an intrinsically disordered protein (IDP) predominantly expressed in the nervous system.^1^ In healthy individuals, α-Syn supports neuronal communication by regulating synaptic vesicle trafficking and neurotransmitter release.^2–4^ However, α-Syn is also implicated in the development of several neurodegenerative diseases, including Parkinson’s disease (PD) and dementia with Lewy bodies.^5–7^ These synucleinopathies are characterized by the abnormal aggregation of α-Syn into fibrils. Although α-Syn fibrils have long been associated with severe neuronal damage, little is known about the molecular processes driving aggregation, which limits our ability to prevent or slow their formation.

The structural and functional properties of α-Syn arise from its three distinct domains. The N-terminal domain (residues 1–60) can fold into a helix or a broken helix. This partially folded state is typically observed upon α-Syn binding to membrane lipids. However, several studies have also reported a partially folded state in solution that would serve as a precursor for α-Syn aggregates.^8,9^ Further, McGlinchey et al. showed that truncating various lengths of the N-terminal domain resulted in decreased fibrillation rates, demonstrating its key role in fibril formation.^10,11^ The second α-Syn domain is the hydrophobic nonamyloid-β component (NAC) domain (residues 61 through 95). The NAC domain is prone to form β-strands, which promote favorable interactions with other NAC domains, stabilizing α-Syn fibrils.^7,12,13^ Finally, the C-terminal domain (residues 96 through 140) contains multiple acidic residues, charged at neutral pH, that will electrostatically repel other α-Syn. This electrostatic repulsion is believed to help regulate α-Syn’s cytotoxicity and preserve its natural unfolded state.^14–16^ Chakroun et al. demonstrated that truncating the C-terminal domain yields faster aggregation compared to full-length α-Syn and even preformed fibrils.^17^

Decades of research have elucidated how factors like ions,^18,19^ pH, temperature,^20^ mutations,^21^ and post-translational modifications^22^ influence the aggregation of α-Syn into fibrils. For example, Munishkina et al. showed that anions induce partial folding in α-Syn and accelerate fibrillation.^23^ The magnitude of the acceleration was also reported to follow the Hofmeister series,^24,25^ which suggests that α-Syn fibrillation occurs through protein–water–anion interactions. In another study, Fujiwara et al. posited that high salt concentrations altered protein dynamics, triggering segmental and local motions that facilitate inter-α-Syn interactions.^26^ Others have reported that an extended α-Syn conformation is necessary for aggregation.^27,28^ This contrasts with the idea advanced by Gorensek-Benitez et al. that since α-Syn is unfolded in its native state in solution, it likely adopts a more compact conformation prior to aggregation.^29^ Nevertheless, researchers largely agree that water dictates the interactions that drive aggregation. In this context, atomistic simulations are indispensable to elucidate how water governs the aggregation propensity of α-Syn. Molecular dynamics (MD) simulations of α-Syn are abundant in the literature and include investigations of its interactions with water^30–32^ at varied salt concentrations.^28,33,34^ Specifically, Stephens et al. used MD to characterize water mobility around a fragment of α-Syn and rationalize aggregation trends in ionic environments.^35^ They find that increasing the concentration of NaCl from 0.15 to 1.5 M increases the nucleation rate of α-Syn, while it decreases the diffusion coefficient of water in both the hydration shell and the bulk. In an independent study, Aggarwal et al. modeled fast- and slow-aggregating α-Syn mutants.^27^ Their findings coincide with those of Stephens et al. in that they observe the fast-aggregating mutants to be less exposed to water, which was characterized by slow reorientation dynamics. Further, they report that the slow-aggregating mutants yield faster translation and rotation water dynamics. They attribute this effect to the increase in overall charge and uptake in hydration waters.^27^ Zacharopoulou also observed an ion-specific decrease in mobility in the hydration shell of α-Syn, which correlates with an increase in aggregation.^28^

Overall, the consensus is that high salt concentrations decrease the mobility of water in the hydration shell of α-Syn, reducing the number of exchanges with bulk water and facilitating interactions with other α-Syn. However, a majority of the MD simulations supporting these findings were performed with nonpolarizable force fields, sometimes on small α-Syn fragments. Many have reported limitations of such force fields for property prediction of water and intrinsically disordered proteins.^36–40^ Herein, we model α-Syn at various NaCl concentrations with the AMOEBA polarizable force field, as it has been shown to accurately account for water–protein interactions.^36,41,42^ We investigate α-Syn in its partially folded amyloidogenic intermediate state (PDB ID: 1XQ8)^43^ to exemplify the role of water in the aggregation process. We reveal a more complex picture than was previously reported, with domain-specific variations in the water mobility.

## Methods

### MD Simulations

The starting protein structure was taken from the protein data bank; PDB ID: 1XQ8.^43^ We used Modeler^44^ to add missing residues and Reduce^45^ to add hydrogen atoms to the structures. We used PACKMOL^46^ to determine the periodic boundary conditions for the protein with a 10 Å buffer on either side, yielding a solvent box size of 92 × 174 × 73 Å^3^. We then used GROMACS^47^ to solvate and neutralize the system. We added Na^+^ and Cl^–^ ions to adjust the systems to [NaCl] = 50, 100, 150, 300, 450, 600, or 900 mM, in addition to a system without excess ions (0 mM). Additionally, we generated solvent boxes without protein at the same [NaCl] to compare how α-Syn hydration shell water properties compare to bulk water as a control. These protein-free water boxes were 70 × 70 × 70 Å^3^.

Each structure was then minimized with the steepest descent, using the AMOEBA polarizable force field^36,41,48^ within the Tinker software package.^49^ MD simulations were run in the NPT ensemble (Bussi thermostat, Montecarlo barostat, 1 fs time step, nonbonded cutoff of 10 Å, Verlet integrator). Simulations were performed at 310 K to replicate physiological temperature as well as at 300 K such that sufficient literature comparisons were available for bulk water property analyses.

Each protein-containing system was run for a total of 350 ns, with frames saved every 10 ps. Equilibration was established within the first 100 ns as monitored by RMSD (Figure S1), with the subsequent 250 ns continued as production simulations. The *xyz* coordinates were saved every nanosecond from 101 to 150 ns to generate 50 new input geometries. These new input geometries were then briefly equilibrated for 10 ps and then continued as production simulations for 20 ps, saving frames every 10 fs to analyze water mobility. Hydration shell waters were defined as water whose oxygen atom was within 3.1 Å of any atom in the protein or its respective subdomain where relevant. For residue-specific analysis, this cutoff distance was dynamically assigned using the peak of the first hydration shell in the radial pair distribution function (Table S1).

The water control simulations were run for 50 ns, with frames saved every 10 ps. *Xyz* coordinates were saved every nanosecond from 26 to 50 ns to generate new input geometries, which were briefly equilibrated for 10 ps and then continued as production simulations for 20 ps, saving frames every 10 fs to analyze water mobility. We used MDAnalysis, with periodic modifications, to perform the analyses described herein.^50,51^ Statistical significance for the below analyses was calculated by a one-way ANOVA

1 $$p=1-F_{\mathrm{CDF}}\left(F_{\mathrm{obs}};{\mathrm{df}}_{\mathrm{between}},{\mathrm{df}}_{\mathrm{total}}\right)$$

where *p* is a measure of significance, *F*_CDF_ is the cumulative probability of *F* up to *F*_obs_ from the ANOVA, df_between_ is the degrees of freedom within each salt concentration (8 salt concentrations – 1 = 7), and df_total_ is the total number of degrees of freedom in the analysis (8 salt concentrations with 50 replicate analyses each = 400–8 salt concentrations = 392). The ANOVA was calculated using the Scipy *F*_oneway_ module.

### Diffusion Coefficient Analysis

The diffusion coefficient (*D*_*i*_) of water was determined by a velocity autocorrelation function (VACF). These were calculated according to eq 2 where *v⃗*_*i*_(*t*_0_) is the velocity of a water molecule at *t* = 0 and *v⃗*_*i*_(*t*_0_ + *t*) is the evolution of that water molecule over time.

2 $$D_{i,\mathrm{VACF}}=\frac{1}{3}\int_{0}^{t_{\max}}\left\langle {\mathrm{v⃗}}_{i}\left(t_{0}\right)\cdot {\mathrm{v⃗}}_{i}\left(t_{0}+t\right)\right\rangle dt$$

The dot product was averaged over all water molecules in a given selection. This was integrated from 0 to *t*_max_ = 1 ps and divided by three to account for spatial dimensionality to yield *D_i_*. The water molecules were selected through the distance criterion described above in the initial frame. The VACF calculation persisted for 1 ps and then repeated again, beginning in the following frame (e.g., trajectory frames 2–101), repeating until there were not enough frames to complete a full ps, and averaging over each replicate within a trajectory. The diffusion coefficients were then reported as the average and standard deviation of the 50 trajectories analyzed per system. All VACF computed in this study are shown in Figures S2–S5.

### Water and Protein Orientation Autocorrelation

The rotational relaxation of water was calculated by a dipole autocorrelation function (DACF)

3 $$C_{\mu ,\mathrm{water}}\left(t\right)=\frac{\left\langle {\mathrm{\mu ̂}}_{i}\left(t_{0}\right)\cdot {\mathrm{\mu ̂}}_{i}\left(t_{0}+t\right)\right\rangle }{\left\langle {\mathrm{\mu ̂}}_{i}\left(t_{0}\right)\cdot {\mathrm{\mu ̂}}_{i}\left(t_{0}\right)\right\rangle }$$

where μ̂_*i*_(*t*_0_) is the unit dipole moment vector of a selected water molecule at *t* = 0, and μ̂_*i*_(*t*_0_ + *t*) is the evolution of that unit dipole moment vector over time. The dot product of these unit vector dipole moments was averaged at each *t* and normalized. The DACF was then fit to a stretched exponential

4 $$C_{\mu }\left(t\right)=e^{{\left(-t/\tau \right)}^{\beta }}$$

where τ is a time constant for the rotational relaxation and β is a factor to describe the complexity or heterogeneity of the exponential decay. For a stretched exponential as described, if β = 1, then the decay can be perfectly described by an exponential decay. If β < 1, then the fit contains multiple contributions to the rotational relaxation.

The water molecules selected were based on distance criteria described above in their initial frame. The DACF calculation persisted for 15 ps and then was repeated again, beginning in the following frame (e.g., trajectory frame 2), repeating until there were not enough frames to complete a full 15 ps analysis, and averaging over each replicate analysis within a trajectory. The τ and β values were then reported as an average and standard deviation of the 50 trajectories analyzed per system. Water exhibits multiple vibrational modes that vary in speed, with fast vibrations (librations) occurring in <1 ps.^52,53^ By omitting the first 2 ps from the raw DACF and renormalizing the data, we can more adequately account for hydrogen bonding interactions between water molecules and water–protein interactions,^54^ and specifically their response to increasing [NaCl]. All DACF computed in this study are shown in Figures S6–S9.

In addition to water reorientation, we also monitor the evolution of the protein reorientation. We measured the protein rotational evolution according to eq 5

5 $$C_{\mu }\left(t\right)=\frac{\left\langle \mathrm{\mu ̂}\left(t_{0}\right)\cdot \mathrm{\mu ̂}\left(t_{0}+t\right)\right\rangle }{\left\langle \mathrm{\mu ̂}\left(t_{0}\right)\cdot \mathrm{\mu ̂}\left(t_{0}\right)\right\rangle }$$

where μ̂(*t*_0_) is the unit dipole moment vector of the protein at a given reference frame and μ­(*t*_0_ + *t*) is the evolution of that unit dipole moment vector over time. The protein dipole moment vector is calculated according to eq 6

6 $$\mathrm{\mu ⃗}\left(t\right)=\sum q_{i}{\mathrm{r⃗}}_{i}\left(t\right)$$

where *q*_*i*_ and *r*_*i*_ are the charge and distance from the protein center of mass for each residue, respectively, at time *t*. This DACF was then fitted to an exponential decay.

### Orientational Tetrahedral Order (OTO) Parameter

The orientational tetrahedral order parameter is a powerful metric to quantify the tetrahedral order of water in a simulation. This parameter is defined by eq 7 ^55,56^

7 $$q_{\mathrm{tet}}=1-\frac{3}{8}\sum_{j=1}^{3}\sum_{k=j+1}^{4}{\left(\cos\left(\psi_{j,k}\right)+\frac{1}{3}\right)}^{2}$$

where ψ_*j*,*k*_ is the angle between the oxygen atom of the *i*th water molecule and its four nearest neighboring water molecule oxygen atoms, *j* and *k*. The average value of all *i*th water molecule oxygen atoms in a given selection yields *q*_tet_, which quantifies the tetrahedral order of the water molecules. For *q*_tet_ = 1, water is considered to have perfect tetrahedral order, with increasing tetrahedral disorder as *q*_tet_ decreases. This measure has previously been used to study water around IDPs, including α-Syn.^27,32,57^

## Results and Discussion

We simulated the dynamics of partially folded, monomeric α-Syn in varied concentrations of NaCl, namely, 0, 50, 100, 150, 300, 450, 600, and 900 mM. Figure 1a shows snapshots of the two limiting cases: 0 and 900 mM. Snapshots for the other concentrations are given in Figure S10. All simulations were performed at physiological (310 K) and ambient (300 K) temperatures to allow further benchmarking against the existing literature.

**Figure 1:**
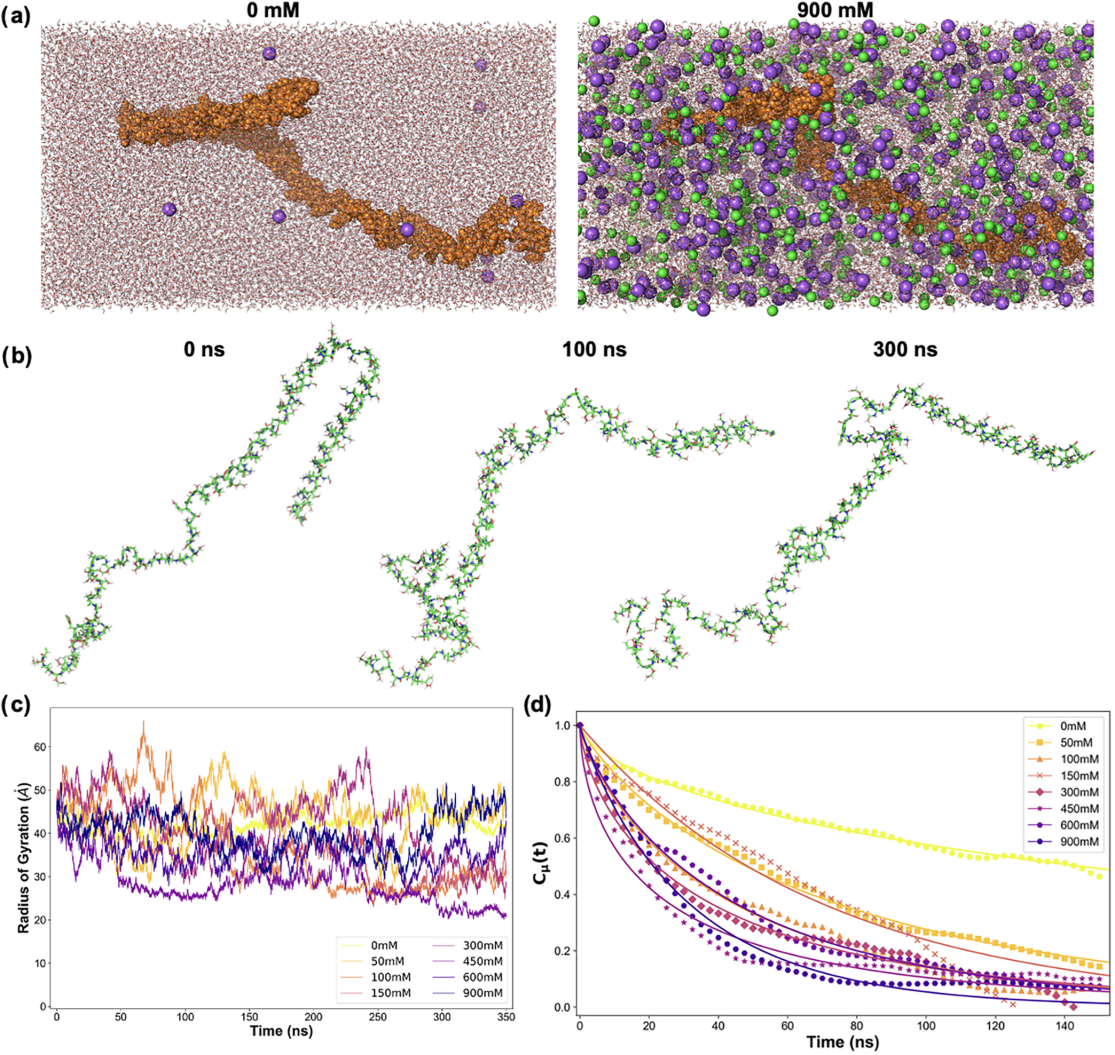
(a) Snapshots of energy minimized systems for NaCl concentrations of 0 (left) and 900 (right) mM. α-Syn is represented in orange, water in red/white, and sodium and chloride ions in purple and green, respectively. (b) Snapshots of α-Syn (water and ions not shown for clarity) at 0, 100, and 300 ns for 900 mM at 310 K. (c) Radius of gyration as a function of time for all the simulations ran at 310 K. Mean and standard deviations are given in Table [Table tblI]. (d) Autocorrelation function of the protein unit dipole moment (*C*_μ_(*t*)) as a function of time and NaCl concentration at 300 K. In each case, the data (symbols) are fitted to a stretched exponential (line). Relaxation times and stretching exponents are given in Table [Table tblII].

### Protein Properties

Figure 1b highlights the structural evolution of α-Syn over the course of simulation with frames taken at 0, 100, and 300 ns for a salt concentration of 900 mM at 310 K. The secondary structure analysis (Figure S11) does not reveal significant changes due to salt concentration although we observe a slight loss of structure when going from 300 to 310 K.

The radius of gyration, *R*_g_, shown in Figure 1c and Table [Table tblI], is similar between the various concentrations at 300 K. However, we observe a reduction in *R*_g_ with salt concentration at 310 K. Since *R*_g_ alone can be difficult to interpret for intrinsically disordered proteins, we also computed the end-to-end distance (*R*_ee_, Table [Table tblI]). Together, our data indicate that NaCl makes α-Syn more compact at both temperatures. Our findings are consistent with the work of Gorensek-Benitez et al., who noted that, unlike globular proteins that must unfold to aggregate, α-Syn is likely more compact in crowded environments, where it aggregates.^29^ Finally, we observe an increase in fluctuations around the average *R*_g_ and *R*_ee_ with salt concentration (Table [Table tblI]). Stronger fluctuations indicate that α-Syn exhibits enhanced local motions in the presence of salt, which has also been recognized as necessary for fibrillation.^16^

**1 tblI:** Mean ± Standard Deviation of the Radius of Gyration (*R*_g_) and End-to-End Distance (*R*_ee_) at 300 and 310 K for the Range of NaCl Concentrations Studied Here

	*R*_g_ (Å)		*R*_ee_ (Å)	
[NaCl] (mM)	300 K	310 K	300 K	310 K
0	42.1 ± 2.4	48.5 ± 2.4	124.4 ± 14.3	140.6 ± 21.7
50	43.5 ± 6.0	36.5 ± 3.9	112.4 ± 22.7	79.0 ± 19.2
100	36.7 ± 10.3	41.1 ± 5.6	95.8 ± 38.8	103.4 ± 18.6
150	37.4 ± 7.2	31.4 ± 6.9	82.6 ± 29.7	75.4 ± 33.4
300	41.6 ± 7.9	34.3 ± 6.0	103.6 ± 40.6	110.7 ± 26.1
450	28.2 ± 4.6	42.0 ± 5.3	29.9 ± 18.5	99.1 ± 24.9
600	35.8 ± 3.3	32.8 ± 6.6	81.1 ± 20.3	60.2 ± 34.1
900	40.2 ± 4.6	33.0 ± 5.2	90.4 ± 29.4	76.6 ± 21.6

We also investigated the influence of the NaCl concentration on the reorientation dynamics of α-Syn. Dey and Biswas have previously shown that protein rotational evolution is well-correlated with fibrillation rates in mutants of the protein fragment amyloid-β.^58^ Specifically, they observed a linear relationship between the computationally derived relaxation constants and experimental fibrillation rates and predicted that protective mutants of amyloid-β demonstrated slower rotational relaxation than causative mutants.^58^ These results were rationalized by considering that faster protein rotational relaxation allows one to sample a greater number of conformations in a given period of time, which may spuriously facilitate the adoption of a fibril-prone state. Here, we followed a similar protocol to characterize the rotational dynamics of α-Syn through the autocorrelation function of the protein unit dipole moment (*C*_μ_ in Figure 1d). After fitting the data to an exponential decay, the data were analyzed; as described in Methods, we present the rotational relaxation constants (τ) in Table [Table tblII].

**2 tblII:** Relaxation Times (τ) for the Autocorrelation Function of the Protein Dipole (*C*_μ_(*t*)) at the Concentrations of NaCl Studied Here[Table-fn tIIfn1]

	300 K		310 K	
[NaCl] (mM)	τ (ns)	*R* ^2^	τ (ns)	*R* ^2^
0	176	0.995	143	0.854
50	74	0.998	61	0.982
100	47	0.991	72	0.638
150	64	0.959	45	0.669
300	41	0.981	41	0.989
450	29	0.983	87	0.896
600	47	0.997	15	0.995
900	32	0.990	41	0.989

a Data for both 300 and 310 K are presented.

We observe that α-Syn rotates slowly without salt but slightly faster at 310 K than 300 K. Additionally, τ drops substantially when salt is introduced, suggesting that α-Syn relaxes faster, in agreement with the findings of Dey and Biswas.^58^ The increased experimental rate of α-Syn fibrillation with increasing salt concentrations, and more specifically the apparent exponential decrease in nucleation rates,^23^ may therefore be explained, in part, by the observed decrease in rotational relaxation constants with increasing NaCl concentration.

### Water Structure

Figure 2 shows the radial distribution functions, *g*(*r*), of α-Syn with water, Na^+^, and Cl^–^ at 310 K (see Figure S12 for those at 300 K and Figures S13 and S14 for the coordination numbers). We observe that the salt concentration does not influence the order or number of water molecules in the vicinity of α-Syn. This is confirmed by computing the number of hydrogen bonds between α-Syn and water, which remains constant between salt concentrations (Figure S15). However, we see that as the salt concentration increases and the number of ions near α-Syn increases, the Na^+^ ions are less ordered. The calculation of *g*(*r*) per domain (Figures S16–S19) reveals that this change in Na^+^ ordering occurs primarily in the C-terminal domain, the most (negatively) charged region in α-Syn. This is consistent with the work of Zacharopoulou et al., who showed that Na^+^ interacts nonspecifically with α-Syn.^28^ In contrast, the interaction pattern of α-Syn with Cl^–^ does not change with the salt concentration.

**Figure 2:**
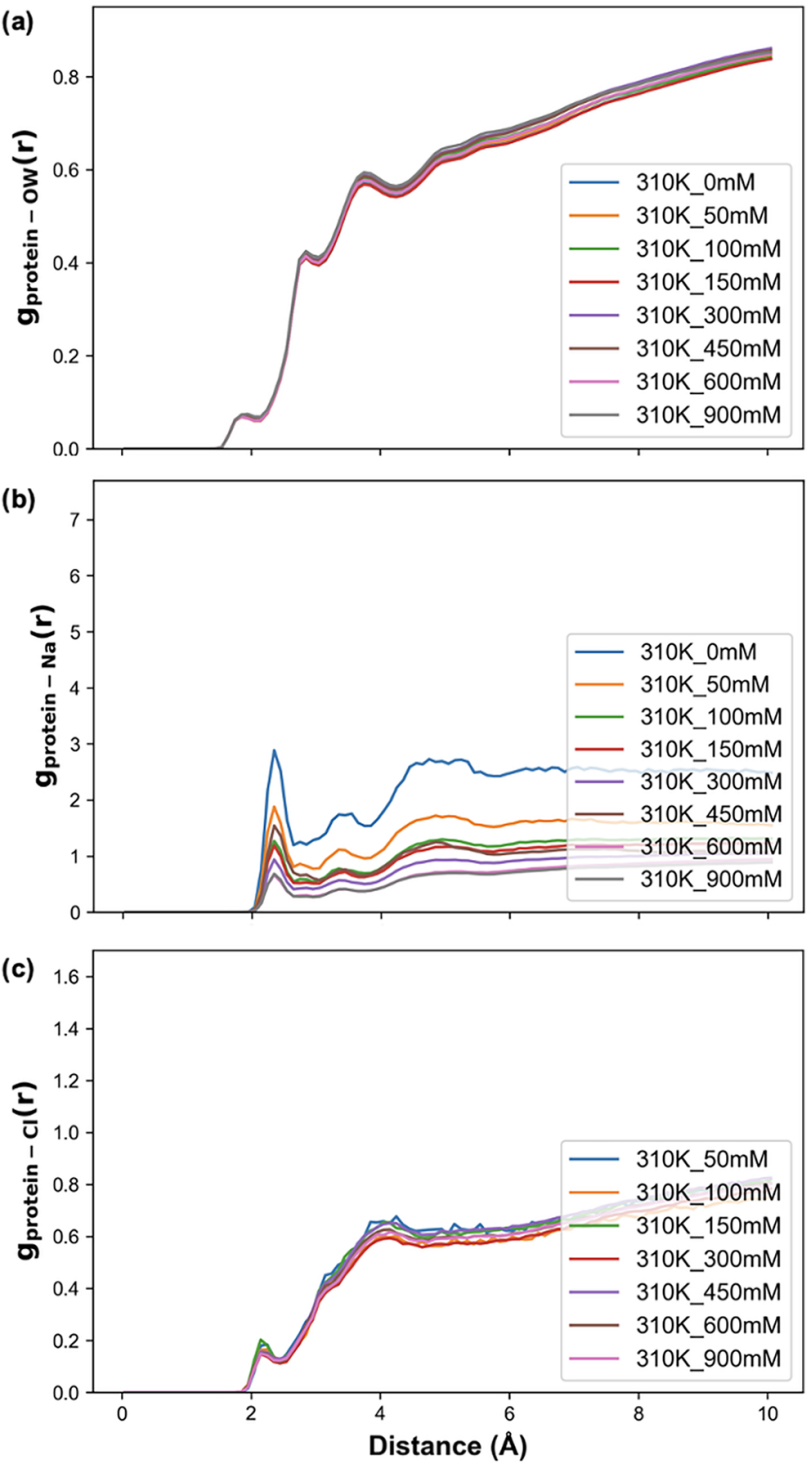
(a) α-Syn-water oxygen radial pair distribution functions for the range of NaCl concentrations at 310 K. (b) α-Syn-Na^+^ radial pair distribution functions for the range of NaCl concentrations at 310 K. (c) α-Syn-Cl^–^ radial pair distribution functions for the range of NaCl concentrations at 310 K. The coordination numbers associated with these distribution functions are shown in Figure S13.

We further characterize the structure of water by computing the Orientational Tetrahedral Order (OTO, *q*_tet_). *q*_tet_ measures how much the local arrangement of water molecules deviates from an ideal tetrahedral geometry. *q*_tet_ = 1 corresponds to perfectly tetrahedral water, while *q*_tet_ = 0 corresponds to completely disordered water. In Figure 3a,b, we show the probability distribution *p*(*q*_tet_) for bulk and hydration shell water, respectively. For bulk water, we observed two maxima at *q*_tet_ = 0.84 and 0.66, indicating the coexistence of water with high and relatively lower orientational order, respectively. Interestingly, while the positions of these populations are minimally impacted by the concentration of NaCl, we observe an increase in the population ratio (Figure 3c). An increased ratio is indicative of a relative increase in bulk water orientational disorder, consistent with the ions disrupting the water hydrogen bond network as their concentration rises. For the water in α-Syn’s hydration shell, we observe a water population with high orientational order centered at the same *q*_tet_ as bulk water (0.84). However, the water population with a relatively lower orientational order shifts to a notably lower *q*_tet_ (0.5), indicating a decreased orientational order for α-Syn hydration shell water. Additionally, we observe a nearly equal population density at these two maxima in the hydration shell, which contrasts with the greater high-ordered water in the bulk. Further, the proportion of relatively lower-to-high orientational order water (*P*(*q*_d_)/*P*(*q*_o_)) increases for all α-Syn systems, compared to bulk water (Figure 3c). However, while *P*(*q*_d_)/*P*(*q*_o_) increased with the concentration of NaCl for bulk water, we did not observe a meaningful trend for the hydration shell waters (i.e., the error bars greatly overlap). Overall, our results suggest that the presence of ions does not significantly disrupt the structure of the hydration shell waters.

**Figure 3:**
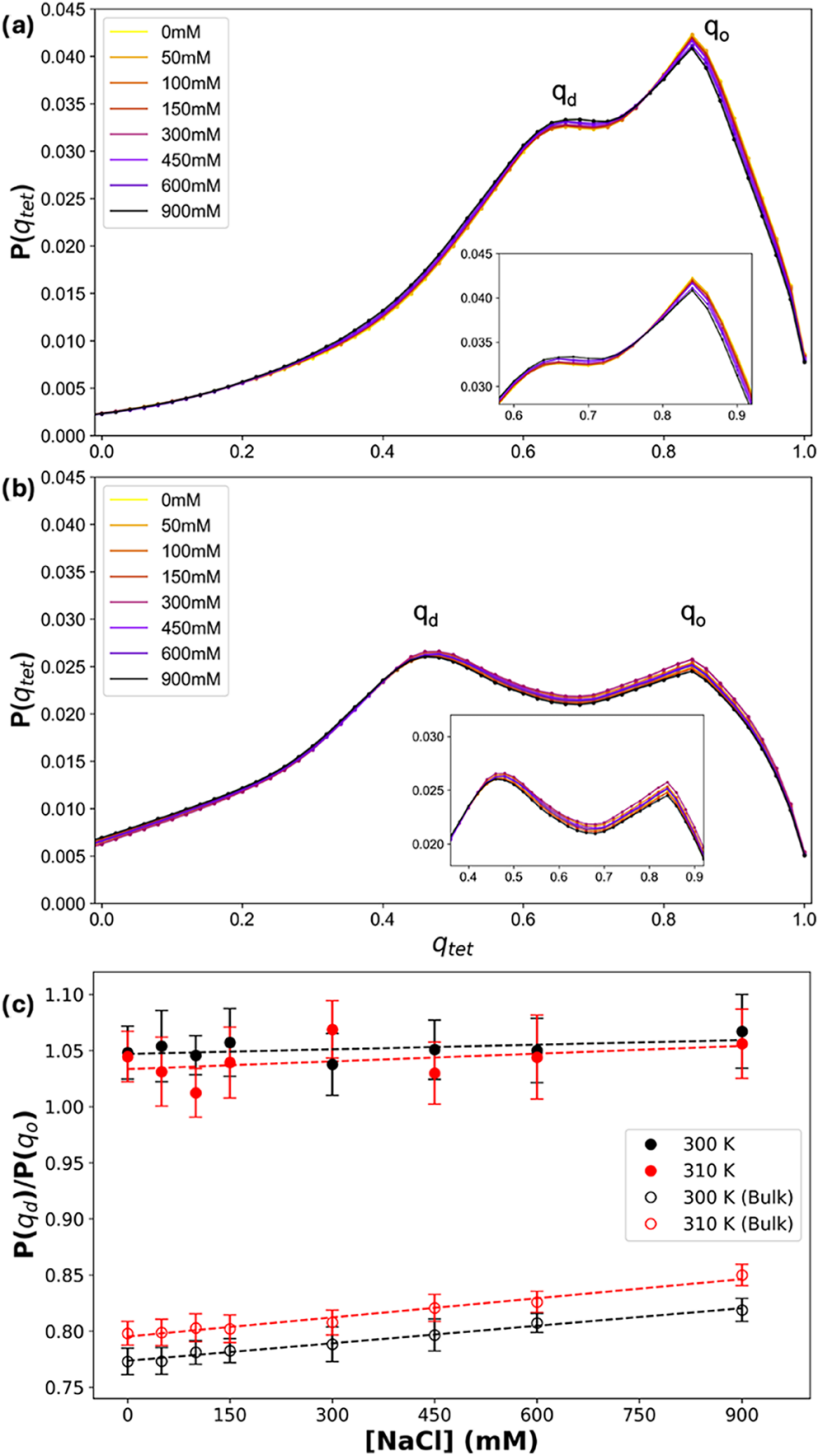
(a) Probability distribution of the OTO parameter for bulk water at different salt concentrations. The maxima for water with high orientational order (*q*_o_) and water with relatively lower orientational order (*q*_d_) are labeled. The inset shows a zoomed view of the two maxima. (b) Probability distribution of the OTO parameter for α-Syn hydration shell water at different salt concentrations. The inset shows a zoomed view of the two maxima. (c) The ratio of relatively lower-to-high orientational order water populations (*P*(*q*_d_)/*P*(*q*_o_)) as a function of NaCl concentration for bulk water (open circles) and α-Syn hydration shell water (closed circles).

### Water Diffusion

We calculated the diffusion coefficient (*D_i_*) of water by integrating the velocity autocorrelation function (VACF), as described in Methods. Figure 4a shows *D_i_* as a function of salt concentration for bulk water (open circles) and hydration shell water (closed circles) at 300 and 310 K. As expected, *D_i_* is consistently lower at 300 K than at 310 K for both bulk and hydration shell water. A similar analysis for each α-Syn subdomain can be seen in Figure S20. *D_i_* is also consistently higher for bulk water than for shell water. At 0 mM, the retardation factor is 2.5 and 2.1 at 300 and 310 K, respectively, consistent with previously published data.^31^

**Figure 4:**
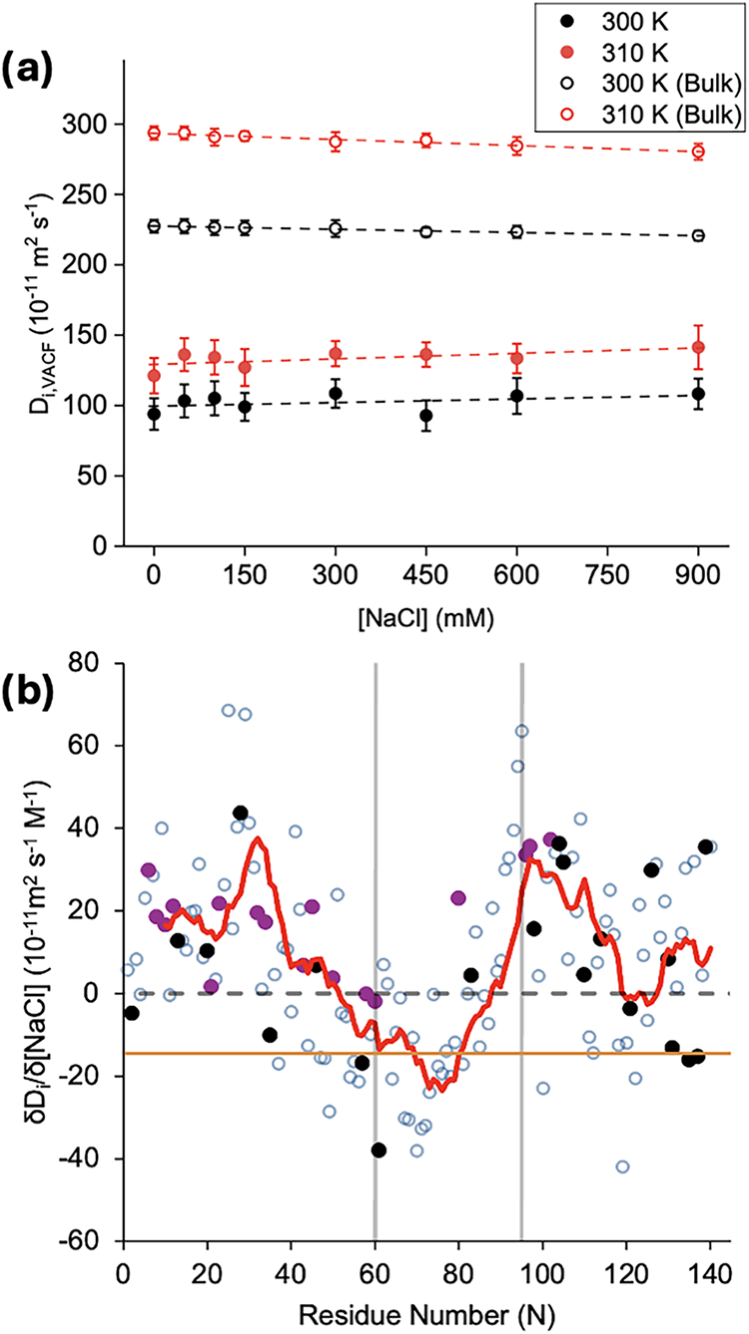
(a) Diffusion coefficient (*D_i_*), computed from VACF, of bulk water (open circles) and α-Syn hydration shell (closed circles) at 300 (black) and 310 K (red). (b) The residue-by-residue slope for the change in *D_i_* as a function of NaCl concentration at 310 K. The red line is a moving average (period 10) of the values. The purple and black data points refer to cationic and anionic residues, respectively. The horizontal dashed line indicates a slope of 0 (i.e., no change in *D_i_* with salt concentration). The orange line indicates the slope of bulk water at 310 K, for reference. The vertical lines at residues 60 and 95 outline the N-terminal, NAC, and C-terminal domains of α-Syn. For reference, the diffusion coefficients per residue at 0 mM are provided in Figure S21.

For bulk water, *D_i_* decreases as the concentration of NaCl increases at a rate (δ*D_i_*/δ*M*_NaCl_) of −7.5 and −14.5 × 10^–11^ m^2^ s^–1^ M_NaCl_^–1^ at 300 and 310 K, respectively. This magnitude of slowdown in the presence of salt agrees well with experiments.^59,60^

In contrast, we see that *D_i_* for hydration shell water increases with an increasing NaCl concentration. At 310 and 300 K, the observed δ*D_i_*/δ*M*_NaCl_ is +5.1 × 10^–11^ m^2^ s^–1^ *M*_NaCl_^–1^ and +4.6 × 10^–11^ m^2^ s^–1^ *M*_NaCl_^–1^, respectively (*p* < 0.05). However, unlike bulk water, the α-Syn hydration shell water is heterogeneous, with more disorder, as shown in Figure 3. The heterogeneity in hydration shell water yields greater standard deviations around the averaged *D_i_*. Since the source of the heterogeneity is the variety of amino acids that make α-Syn, we calculated the rate of change of *D_i_* for hydration water per residue (Figure 4b). 124 and 131 residues out of 140 were found to exhibit statistically significant δ*D_i_*/δ*M*_NaCl_ (*p* < 0.05) at 300 and 310 K, respectively. This residue-specific analysis enabled us to identify domain-dependent variations in *D_i_* for hydration shell waters. Notably, the NAC domain demonstrated primarily negative rates, which contrast with those of both the N- and C-terminal domains. A recent study found that the diffusion coefficient of the α-Syn hydration shell and the bulk water decreased when the NaCl concentration increased from 0.15 to 1.5 M.^35^ Since they specifically analyzed a truncated representation of α-Syn (residues 72–78 in the NAC domain), their observed decrease in mobility agrees with our results for the same segment. However, we see here that the decreased water mobility does not generalize to the whole α-Syn. Indeed, residues in the N- and C-terminal domains exhibit hydration shell waters with enhanced diffusion kinetics, suggesting a higher exchange rate with the bulk, similar to a partial dehydration of α-Syn. Interestingly, K96 and K97, which are implicated in fibrillation,^61^ exhibit the highest increase in water mobility with increasing salt concentration. Prior studies also show that deleting residues 9–30 increases aggregation, whereas removing residues 36–57 reduces it.^62^ Truncating the C-terminus beyond residue 120 likewise accelerates aggregation.^63^ Taken with our data, these trends point to two regimes: (i) excising segments that display fast water-diffusion kinetics impede aggregation, while (ii) trimming the head and tail of the N- or C-terminal domains amplifies aggregation by exposing the more centrally located fast water-diffusion kinetics residues to bulk solvent. This increased exposure to water with greater mobility in response to increasing salt concentration results in greater exchange between hydration shell and bulk water, hastening dehydration, and lowers the barrier to intermolecular contacts. Meanwhile, the NAC domain engages more strongly with the surrounding water relative to 0 mM, as evidenced by its decreased mobility with increasing salt concentration. These slow waters mediate bridges between neighboring NAC segments and stabilize fibrils.

### Water Reorientation

Finally, we characterized the rotational dynamics of water using the dipole autocorrelation function (DACF) that we fitted to a stretched exponential (see Methods). In Figure 5a, we show the relaxation time constant, τ, as a function of salt concentration for bulk and hydration shell water at 300 and 310 K. For bulk water, we first observe τ to be smaller at 310 K than at 300 K for all concentrations. This decrease in τ is consistent with faster dynamics, expected at a higher temperature. Second, we see that τ increased with salt concentration, indicating that bulk water rotates more slowly at higher salt concentrations. Both of these observations agree with the literature.^64,65^ Similarly to what was observed for the diffusion coefficient, the relaxation dynamics of hydration shell waters demonstrated an opposite trend from bulk water. At 0 mM, we observe a retardation factor for the hydration shell water of 3.4 and 2.8 at 300 and 310 K, respectively. τ then decreases with increasing NaCl concentration at a rate of −0.56 and −0.51 ps *M*_NaCl_^–1^ for 300 and 310 K, respectively (*p* < 0.05).

**Figure 5:**
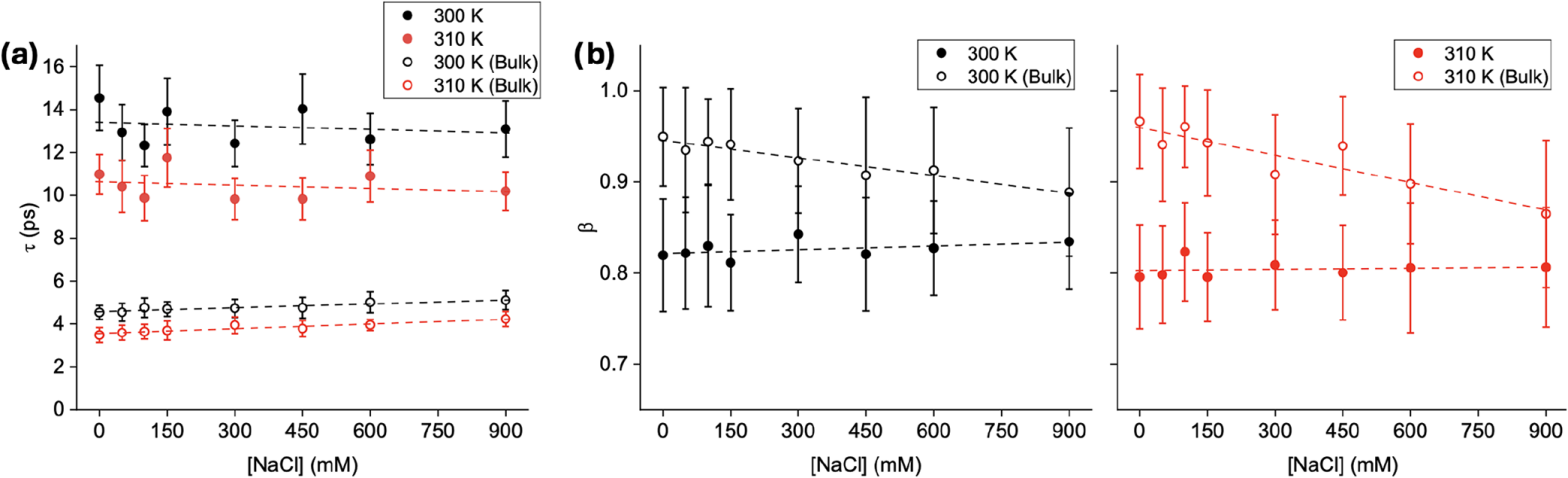
(a) Rotational relaxation constant (τ) of bulk water (open circles) and the α-Syn hydration shell (closed circles) at 300 K (black) and 310 K (red) at different [NaCl]. τ was computed by fitting a stretched exponential to the DACF data. (b) The stretching exponent (β) for the DACF of bulk and hydration shell waters at 300 K (left) and 310 K (right) at different concentrations of NaCl.

The stretching exponent, β, also varies significantly for bulk and hydration shell water (Figure 5b). At 0 mM, β ≈ 0.95 for bulk water indicated a quasiexponential behavior. However, β decreases with increasing NaCl concentration, suggesting more complex relaxation dynamics consistent with enhanced water–ion interactions. For the hydration shell water, we observe lower values of β at all concentrations, reflecting the fact that rotational dynamics results from a combination of water–water and water–protein interactions. However, unlike for diffusion, no significant trend was observed with respect to the NaCl concentration (*p* > 0.05 for β). The lack of dependence on salt concentration suggests that the rotational dynamics of the hydration shell water is still dominated by protein–water interactions and not salt–water interactions.

## Conclusion

We used polarizable MD to investigate the influence of salt, a promoter of α-Syn fibrillation, on the hydration shell water structure and dynamics. We find that salt disrupts the local arrangement of bulk water molecules, but not that of hydration shell water. This is likely because the structure of the hydration shell water is governed by its interaction with α-Syn. Nevertheless, we find that water dynamics were impacted by the presence of salt. For bulk, the diffusion coefficient decreased with increasing NaCl concentration. For hydration waters, we find the opposite trend. A detailed analysis of the diffusion coefficient per residue in α-Syn revealed that hydration water in the N-terminal and C-terminal domains diffuses faster as salt concentration increases, while hydration waters in the NAC domain slow down. The slowdown in the NAC domain corroborates previous findings for the same region^35^ and replicates what is observed in bulk water. However, our work uncovers a more complex picture where waters in the two domains known to regulate α-Syn fibrillation accelerate instead of slowing down with salt concentration. Interestingly, we did not observe a change in the rotational mobility of hydration shell water with salt. Overall, our work suggests that intermolecular interactions between α-Syn would be promoted by partial dehydration of residues in the N- and C-terminal domains, particularly those previously reported to regulate fibrillation. This partial dehydration of the N- and C-terminal domains is accompanied by stronger protein–water interactions in the NAC domain, which could help to bridge proteins into fibrils. Future work will involve investigating different ions such as SO_4_^2–^ and ^–^SCN to probe how more chaotropic or kosmotropic ions influence the relative mobility of hydration shell water.

## Supplementary Material



## Notes

Additional data and associated explanations: (i) RMSD, (ii) VACF and DACF, (iii) MD snapshots, (iv) secondary structure evolution, (v) radial pair distribution and coordination numbers for water and ions with protein, (vi) hydrogen bond counts, (vii) domain-specific comparisons of protein–Na^+^ radial pair distribution functions and coordination numbers, and (viii) domain-specific salt-influenced hydration shell water diffusion coefficients (PDF)

## References

[1] Weinreb P. H., Zhen W., Poon A., Conway K., Lansbury P. (1996). NACP, A Protein
Implicated in Alzheimer’s Disease and Learning, Is Natively
Unfolded. Biochemistry.

[2] Burré J., Sharma M., Tsetsenis T., Buchman V., Etherton M., Südhof T. (2010). α-Synuclein
Promotes SNARE-Complex Assembly in
Vivo and in Vitro. Science.

[3] Burré J., Sharma M., Südhof T. (2014). α-Synuclein
assembles into
higher-order multimers upon membrane binding to promote SNARE complex
formation. Proc. Natl. Acad. Sci. U.S.A..

[4] Pranke I. M., Morello V., Bigay J., Gibson K., Verbavatz J.-M., Antonny B., Jackson C. (2011). α-Synuclein and
ALPS motifs
are membrane curvature sensors whose contrasting chemistry mediates
selective vesicle binding. J. Cell Biol..

[5] Spillantini M. G., Shmidt M., Lee V.-Y., Trojanowski J., Jakes R., Goedert M. (1997). α-Synuclein in
Lewy bodies. Nature.

[6] Spillantini M. G., Crowther R., Jakes R., Hasegawa M., Goedert M. (1998). α-Synuclein
in filamentous inclusions of Lewy bodies from Parkinson’s disease
and dementia with Lewy bodies. Proc. Natl. Acad.
Sci. U.S.A..

[7] Ke P. C., Zhou R., Serpell L., Riek R., Knowles T., Lashuel H., Gazit E., Hamley I., Davis T., Fändrich M., Otzen D., Chapman M., Dobson C., Eisenberg D., Mezzenga R. (2020). Half a century of amyloids: past,
present and future. Chem. Soc. Rev..

[8] Sode K., Ochiai S., Kobayashi N., Usuzaka E. (2007). Effect of Reparation
of Repeat Sequences in the Human α-Synuclein on Fibrillation
Ability. Int. J. Biol. Sci..

[9] Bartels T., Ahlstrom L., Leftin A., Kamp F., Haass C., Brown M., Beyer K. (2010). The N-Terminus
of the Intrinsically
Disordered Protein α-Synuclein Triggers Membrane Binding and
Helix Folding. Biophys. J..

[10] McGlinchey R. P., Ni X., Shadish J., Jiang J., Lee J. (2021). The N terminus of -synuclein
dictates fibril formation. Proc. Natl. Acad.
Sci. U.S.A..

[11] McGlinchey R. P., Ramos S., Dimitriadis E. K., Wilson C. B., Lee J. C. (2025). Defining
essential charged residues in fibril formation of a lysosomal derived
N-terminal α-synuclein truncation. Nat.
Commun..

[12] Li B., Ge P., Peng G., Murray K., Sheth P., Zhang M., Nair G., Sawaya M., Shin W., Boyer D., Ye S., Esenberg D., Eisenberg D. S., Zhou Z. H., Jiang L. (2018). Cryo-EM of
full-length α-synuclein reveals fibril polymorphs with a common
structural kernel. Nat. Commun..

[13] Giasson B. I., Murray I., Trojanowski J., Lee V.-Y. (2001). A Hydrophobic Stretch
of 12 Amino Acid Residues in the Middle of α-Synuclein Is Essential
for Filament Assembly. J. Biol. Chem..

[14] Zhang C., Pei Y., Zhang Z., Xu L., Lie X., Jiang L., Pielak G., Zhou X., Liu M., Li C. (2022). C-terminal
truncation modulates α-Synuclein’s cytotoxicity and aggregation
by promoting the interactions with membrane and chaperone. Commun. Biol..

[15] Farzadfard A., Pedersen J., Meisl G., Somavarapu A., Alam P., Goksoyr L., Nielsen M., Sander A., Knowles T., Pedersen J., Otzen D. (2022). The C-terminal
tail
of α-synuclein protects against aggregate replication but is
critical for oligomerization. Commun. Biol..

[16] Croke R. L., Sallum C. O., Watson E., Watt E. D., Alexandrescu A. T. (2008). Hydrogen
exchange of monomeric α-synuclein shows unfolded structure persists
at physiological temperature and is independent of molecular crowding
in *Escherichia coli*. Protein Sci..

[17] Chakroun T., Evsyukov V., Nykänen N.-P., Höllerhage M., Schmidt A., Kamp F., Ruf V., Wurst W., Rösler T., Höglinger G. (2020). Alpha-synuclein fragments trigger
distinct aggregation pathways. Cell Death Dis..

[18] Uversky V., Li J., Fink A. (2001). Metal-triggered Structural
Transformations, Aggregation,
and Fibrillation of Human α-Synuclein: A possible molecular
link between parkinsons disease and heavy metal exposure. J. Biol. Chem..

[19] Han J. Y., Choi T., Kim H. (2018). Molecular Role of Ca2+ and Hard Divalent
Metal Cations on Accelerated Fibrillation and Interfibrillar Aggregation
of α-Synuclein. Sci. Rep..

[20] Uversky V. N., Li J., Fink A. (2001). Evidence for a Partially
Folded Intermediate in α-Synuclein
Fibril Formation. J. Biol. Chem..

[21] Ruf V. C., Nübling G., Willikens S., Shi S., Schmidt F., Levin J., Bötzel K., Kamp F., Giese A. (2019). Different
Effects of α-Synuclein Mutants on Lipid Binding and Aggregation
Detected by Single Molecule Fluorescence Spectroscopy and ThT Fluorescence-Based
Measurements. ACS Chem. Neurosci..

[22] Zhang J., Li X., Li J.-D. (2019). The Roles
of Post-translational Modifications on α-Synuclein
in the Pathogenesis of Parkinson’s Diseases. Front. Neurosci..

[23] Munishkina L. A., Henriques J., Uversky V., Fink A. (2004). Role of ProteinWater
Interactions and Electrostatics in α-Synuclein Fibril Formation. Biochemistry.

[24] Hofmeister F. (1888). Zur Lehre
von der Wirkung der Salze. Arch. Exp. Pathol.
Pharmakol..

[25] Zhang Y., Cremer P. (2010). Chemistry of Hofmeister
Anions and Osmolytes. Annu. Rev. Phys. Chem..

[26] Fujiwara S., Kono F., Matsuo T., Sugimoto Y., Matsumoto T., Narita A., Shibata K. (2019). Dynamic properties
of human α-synuclein
related to propensity to amyloid fibril formation. J. Mol. Biol..

[27] Aggarwal L., Karmakar S., Biswas P. (2024). Differentially heterogeneous
hydration
environment of the familial mutants of α-synuclein. J. Chem. Phys..

[28] Zacharopoulou M., Seetaloo N., Ross J., Stephens A. D., Fusco G., McCoy T. M., Dai W., Mela I., Fernandez-Villegas A., Martel A., Routh A. F., De Simone A., Phillips J. J., Schierle G. S. K. (2025). Local Ionic Conditions
Modulate the
Aggregation Propensity and Influence the Structural Polymorphism of
α-Synuclein. J. Am. Chem. Soc..

[29] Gorensek-Benitez A. H., Kirk B., Myers J. K. (2022). Protein fibrillation
under crowded
conditions. Biomolecules.

[30] Hazy E., Bokor M., Kalmar L., Gelencser A., Kamasa P., Han K.-H., Tompa K., Tompa P. (2011). Distinct Hydration
Properties of Wild-Type and Familial Point Mutant A53T of α-Synuclein
Associated with Parkinson’s Disease. Biophys. J..

[31] Rani P., Biswas P. (2015). Diffusion of Hydration Water around Intrinsically Disordered
Proteins. J. Phys. Chem. B.

[32] Rani P., Biswas P. (2015). Local Structure and
Dynamics of Hydration Water in
Intrinsically Disordered Proteins. J. Phys.
Chem. B.

[33] Ramis R., Ortega-Castro J., Vilanova B., Adrover M., Frau J. (2020). Unraveling
the NaCl Concentration Effect on the First Stages of α-Synuclein
Aggregation. Biomacromolecules.

[34] Imaura R., Kawata Y., Matsuo K. (2024). Salt-Induced
Hydrophobic C-Terminal
Region of α-Synuclein Triggers Its Fibrillation under the Mimic
Physiologic Condition. Langmuir.

[35] Stephens A. D., Kölbel J., Moons R., Chung C., Ruggiero M., Mahmoudi N., Shmool T., McCoy T., Nietlispach D., Routh A., Sobott F., Zeitler J., Schierle G. S. K. (2023). Decreased
Water Mobility Contributes To Increased α-Synuclein Aggregation. Angew. Chem., Int. Ed..

[36] Ponder J. W., Wu C., Ren P., Pande V., Chodera J., Schnieders M., Haque I., Mobley D., Lambrecht D., DiStasio R. J., Head-Gordon M., Clark G., Johnson M., Head-Gordon T. (2010). Current Status of the Amoeba Polarizable Force Field. J. Phys. Chem. B.

[37] Henriques J., Cragnell C., Skepo M. (2015). Molecular dynamics simulations of
intrinsically disordered proteins: force field evaluation and comparison
with experiment. J. Chem. Theory Comput..

[38] Inakollu V. S., Geerke D. P., Rowley C. N., Yu H. (2020). Polarisable force fields:
what do they add in biomolecular simulations?. Curr. Opin. Struct. Biol..

[39] Huang J., MacKerell A. D. (2018). Force field development
and simulations
of intrinsically disordered proteins. Curr.
Opin. Struct. Biol..

[40] Wang A., Zhang Z., Li G. (2018). Higher accuracy achieved
in the simulations
of protein structure refinement, protein folding, and intrinsically
disordered proteins using polarizable force fields. J. Phys. Chem. Lett..

[41] Laury M. L., Wang L.-P., Pande V., Head-Gordon T., Ponder J. (2015). Revised Parameters for the Amoeba Polarizable Atomic
Multipole Water Model. J. Phys. Chem. B.

[42] Bradshaw R. T., Dziedzic J., Skylaris C.-K., Essex J. W. (2020). The role of electrostatics
in enzymes: do biomolecular force fields reflect protein electric
fields?. J. Chem. Inf. Model..

[43] Ulmer T. S., Bax A., Cole N., Nussbaum R. (2005). Structure and Dynamics of Micelle-bound
Human α-Synuclein. J. Biol. Chem..

[44] Webb B., Sali A. (2016). Comparative Protein
Structure Modeling Using modeller. Curr. Protoc.
Protein Sci..

[45] Word J., Lovell S., Richardson J., Richardson D. (1999). Asparagine
and glutamine: using hydrogen atom contacts in the choice of side-chain
amide orientation. J. Mol. Biol..

[46] Martínez L., Andrade R., Birgin E., Martínez J. (2009). Packmol: A
package for building initial configurations for molecular dynamics
simulations. J. Comput. Chem..

[47] Van
Der Spoel D., Lindahl E., Hess B., Groenhof G., Mark A., Berendsen H. J. (2005). Gromacs: Fast, flexible, and free. J. Comput. Chem..

[48] Zhang C., Lu C., Jing Z., Wu C., Piquemal J.-P., Ponder J., Ren P. (2018). Amoeba Polarizable Atomic Multipole Force Field for Nucleic Acids. J. Chem. Theory Comput..

[49] Harger M., Li M., Wang Z., Dalby K., Lagardère L., Piquemal J.-P., Ponder J., Ren P. (2017). Tinker-OpenMM:
Absolute
and relative alchemical free energies using Amoeba on GPUs. J. Comput. Chem..

[50] Michaud-Agrawal N., Denning E., Woolf T., Beckstein O. (2011). MDAnalysis:
A toolkit for the analysis of molecular dynamics simulations. J. Comput. Chem..

[51] Gowers, R. ; Linke, M. ; Barnoud, J. ; Reddy, T. ; Melo, M. ; Seyler, S. ; Domański, J. ; Dotson, D. ; Buchoux, S. ; Kenney, I. ; Beckstein, O. In MDAnalysis: A Python Package for the Rapid Analysis of Molecular Dynamics Simulations, Proceedings of the 15th Python in Science Conference, 2016; pp 98–105.

[52] Laage D., Stirnemann G., Sterpone F., Rey R., Hynes J. (2011). Reorientation
and Allied Dynamics in Water and Aqueous Solutions. Annu. Rev. Phys. Chem..

[53] Laage D., Hynes J. (2008). On the Molecular Mechanism
of Water Reorientation. J. Phys. Chem. B.

[54] Fogarty A. C., Laage D. (2014). Water Dynamics in Protein
Hydration Shells: The Molecular Origins
of the Dynamical Perturbation. J. Phys. Chem.
B.

[55] Chau P.-L., Hardwick A. (1998). A new order parameter
for tetrahedral configurations. Mol. Phys..

[56] Errington J. R., Debenedetti P. (2001). Relationship
between structural order and the anomalies
of liquid water. Nature.

[57] Aggarwal L., Biswas P. (2018). Hydration Water Distribution
around Intrinsically Disordered
Proteins. J. Phys. Chem. B.

[58] Dey P., Biswas P. (2023). Relaxation dynamics
measure the aggregation propensity
of amyloid-ß and its mutants. J. Chem.
Phys..

[59] Müller K. J., Hertz H. (1996). A Parameter as an Indicator for WaterWater Association in Solutions
of Strong Electrolytes. J. Phys. Chem. A.

[60] Tanaka K. (1975). Measurements
of self-diffusion coefficients of water in pure water and in aqueous
electrolyte solutions. J. Chem. Soc., Faraday
Trans. 1.

[61] McGlinchey R. P., Ramos S., Dimitriadis E., Wilson C., Lee E. (2025). Defining essential
charged residues in fibril formation of a lysosomal derived N-terminal
α-synuclein truncation. Nat. Commun..

[62] Huang F., Yan J., Xu H., Wang Y., Zhang X., Zou Y., Lian J., Ding F., Sun Y. (2024). Exploring the impact
of physiological C-Terminal truncation on α-Synuclein conformations
to unveil mechanisms regulating pathological aggregation. J. Chem. Inf. Model..

[63] Terada M., Suzuki G., Nonaka T., Kametani F., Tamaoka A., Hasegawa M. (2018). The effect of truncation on prion-like
properties of
α-synuclein. J. Biol. Chem..

[64] Endom L., Hertz H., Thül B., Zeidler M. (1967). A Microdynamic Model
of Electrolyte Solutions as Derived from Nuclear Magnetic Relaxation
and Self-Diffusion Data. Ber. Bunsenges. Phys.
Chem..

[65] Stirnemann G., Wernersson E., Jungwirth P., Laage D. (2013). Mechanisms of Acceleration
and Retardation of Water Dynamics by Ions. J.
Am. Chem. Soc..

