# Supplementary material for: Water Accelerates in the Hydration Shell of the N- and C‑Terminal Domains of α‑Synuclein in the Presence of NaCl
Source: J Phys Chem B. 2026 Jan 21;130(4):1148–56. doi: 10.1021/acs.jpcb.5c06647 (PMC12862797; doi:10.1021/acs.jpcb.5c06647)
Supplement: Supplementary file 1 [file jp5c06647_si_001.pdf]

Water Accelerates in the Hydration Shell of the N- and C-Terminal Domains of α-Synuclein in the Presence of NaCl

Stephen J. Koehler[†,‡] and Valerie Vaissier Welborn[*,†,‡]

†*Department of Chemistry, Virginia Tech, Blacksburg, VA 24060, USA*

‡*Macromolecules Innovation Institute (MII),Virginia Tech, Blacksburg, VA 24060, USA*

E-mail: vwelborn@vt.edu

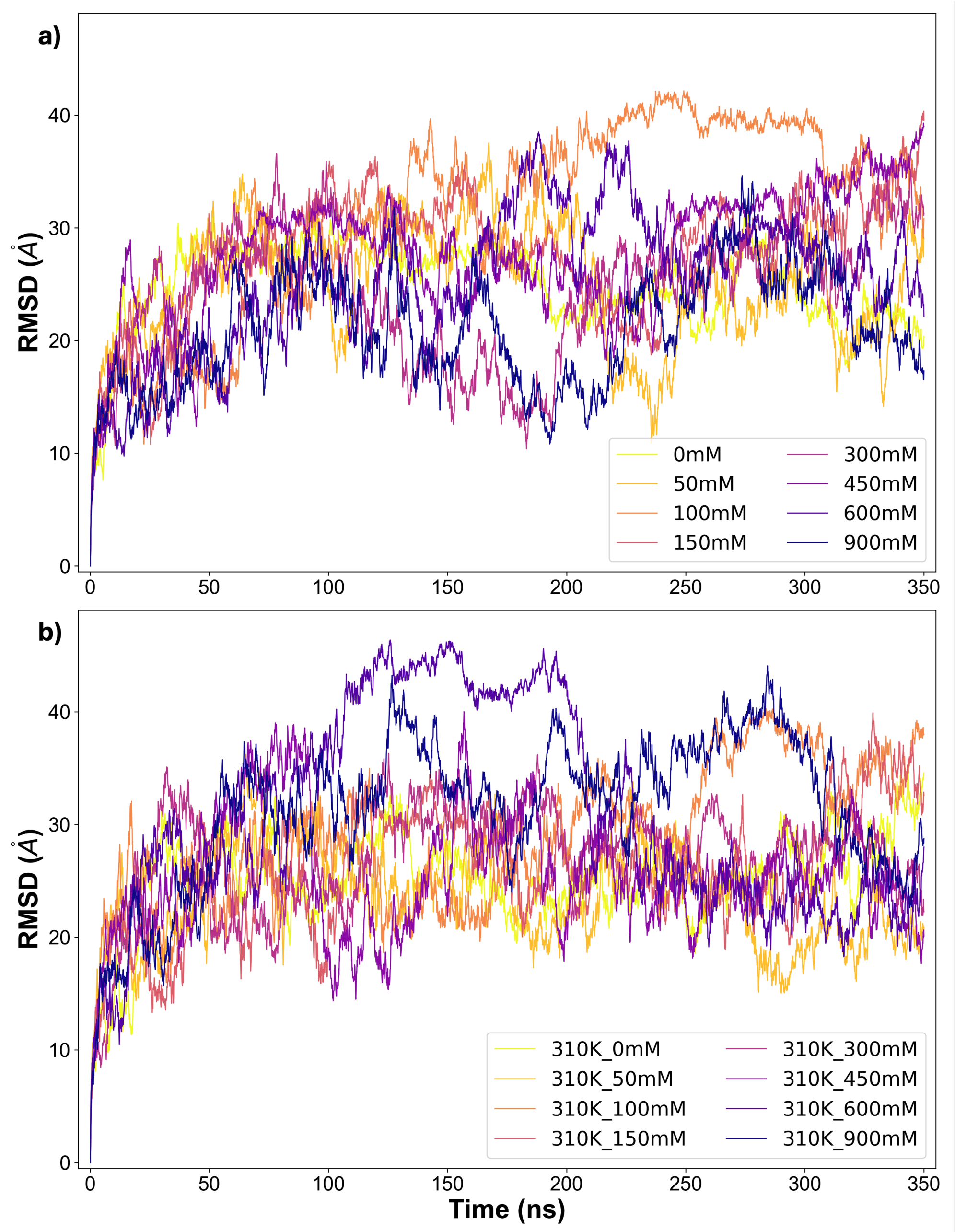

Figure S1: Root mean squared deviation (RMSD) for the 8 different NaCl concentrations investigated at a) 300 K and b) 310 K.

Table S1: Cutoff distance d (Å) of α-Syn hydration shell used for residue-selective N analyses.

N	d (Å)	N	d (Å)	N	d (Å)	N	d (Å)
1	3.0	36	3.2	71	3.4	106	3.1
2	3.2	37	3.3	72	3.3	107	3.2
3	3.2	38	3.3	73	3.4	108	3.5
4	3.4	39	3.3	74	3.3	109	3.2
5	3.3	40	3.3	75	3.3	110	3.2
6	3.1	41	3.1	76	3.4	111	3.1
7	3.4	42	3.1	77	3.3	112	3.2
8	3.4	43	3.1	78	3.4	113	3.2
9	3.3	44	3.1	79	3.3	114	3.2
10	3.1	45	3.1	80	3.2	115	3.2
11	3.3	46	3.2	81	3.2	116	3.3
12	3.1	47	3.1	82	3.3	117	3.4
13	3.3	48	3.3	83	3.3	118	3.2
14	3.4	49	3.3	84	3.3	119	3.2
15	3.3	50	3.2	85	3.3	120	3.4
16	3.4	51	3.2	86	3.2	121	3.2
17	3.4	52	3.3	87	3.1	122	3.3
18	3.3	53	3.2	88	3.2	123	3.1
19	3.4	54	3.2	89	3.1	124	3.2
20	3.4	55	3.4	90	3.1	125	3.3
21	3.1	56	3.4	91	3.1	126	3.2
22	3.3	57	3.4	92	3.1	127	3.4
23	3.2	58	3.1	93	3.1	128	3.4
24	3.3	59	3.3	94	3.3	129	3.1
25	3.4	60	3.2	95	3.2	130	3.3
26	3.3	61	3.4	96	3.1	131	3.3
27	3.4	62	3.3	97	3.1	132	3.2
28	3.3	63	3.4	98	3.2	133	3.4
29	3.4	64	3.3	99	3.2	134	3.2
30	3.4	65	3.3	100	3.3	135	3.2
31	3.4	66	3.4	101	3.1	136	3.4
32	3.1	67	3.4	102	3.2	137	3.2
33	3.2	68	3.4	103	3.1	138	3.4
34	3.2	69	3.3	104	3.2	139	3.1
35	3.4	70	3.4	105	3.1	140	3.2

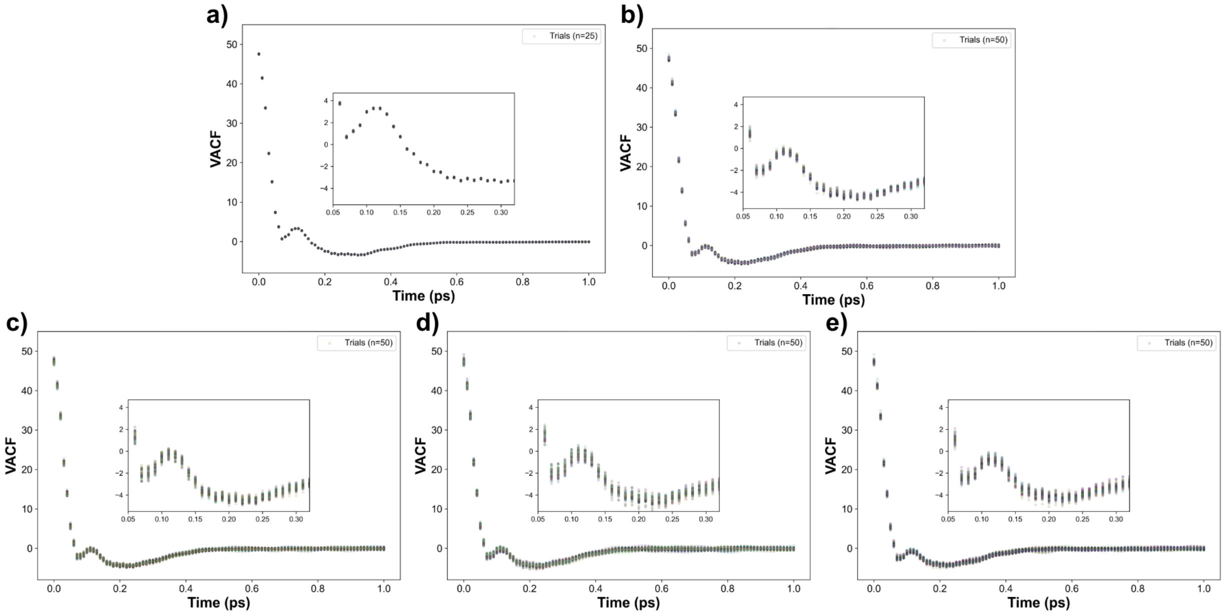

Figure S2: Raw VACF data at 0 mM and 300K for bulk water (a) and hydration shell water for α-Syn (b), the N-Terminal domain (c), NAC domain (d), and C-Terminal domain (e). Each plot shows the every replicate simulation's data overlayed (n=25 for bulk, n=50 for hydration shell water).

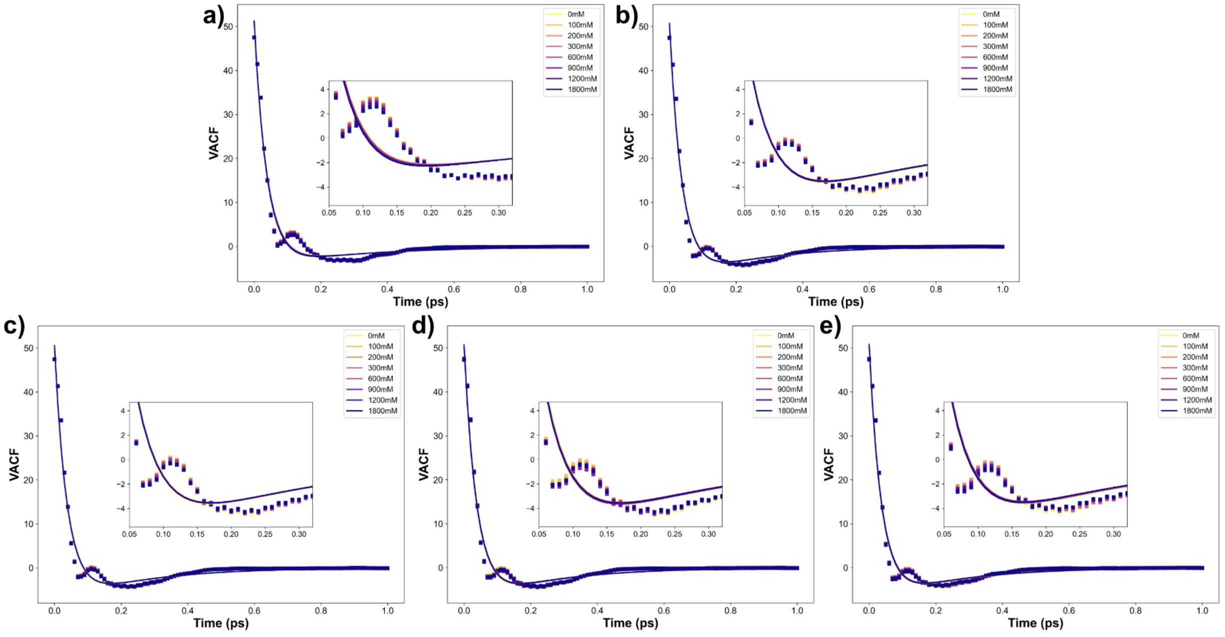

Figure S3: Averaged VACF data of each replicate simulation for each salt concentration tested at 300K for bulk water (a) and hydration shell water for α-Syn (b), the N-Terminal domain (c), NAC domain (d), and C-Terminal domain (e). Each set of averaged data is accompanied by a double exponential fit, and also includes an inset to help elucidate subtle differences between the various salt concentrations.

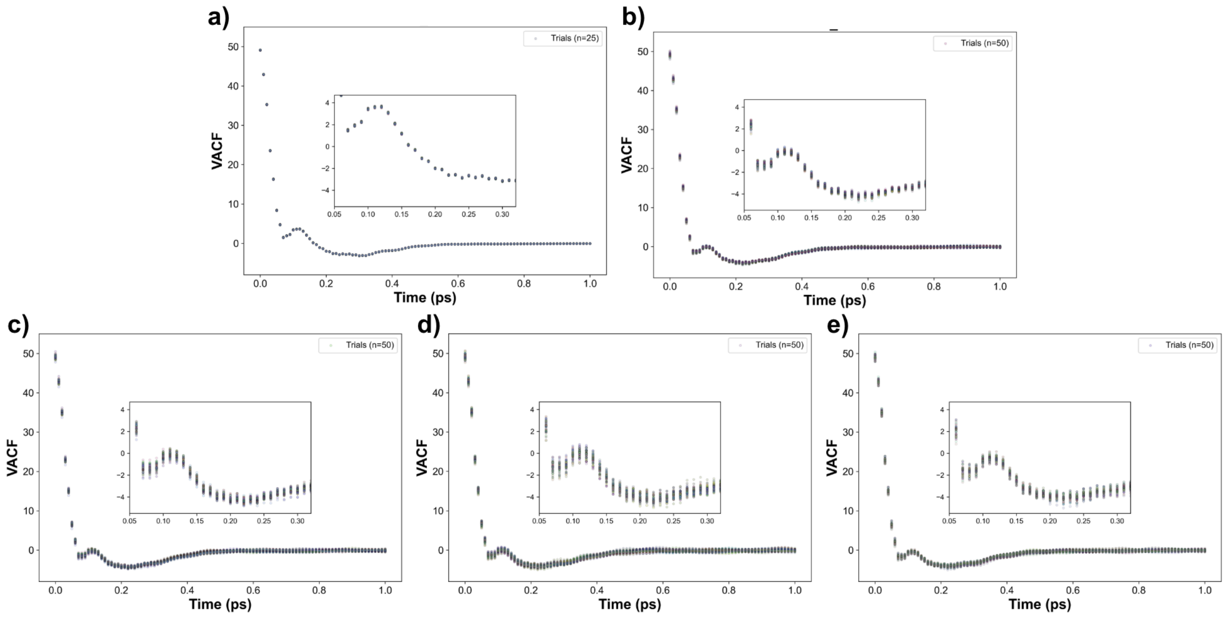

Figure S4: Raw VACF data at 0 mM and 310K for bulk water (a) and hydration shell water for α-Syn (b), the N-Terminal domain (c), NAC domain (d), and C-Terminal domain (e). Each plot shows the every replicate simulation's data overlayed (n=25 for bulk, n=50 for hydration shell water).

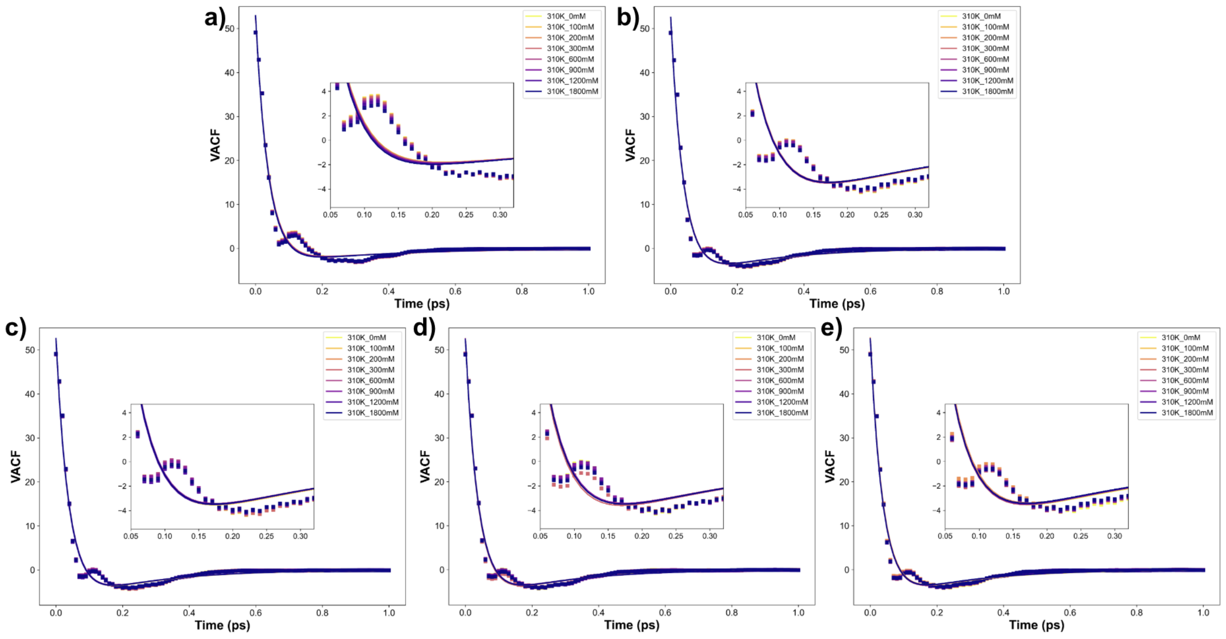

Figure S5: Averaged VACF data of each replicate simulation for each salt concentration tested at 310K for bulk water (a) and hydration shell water for α-Syn (b), the N-Terminal domain (c), NAC domain (d), and C-Terminal domain (e). Each set of averaged data is accompanied by a double exponential fit, and also includes an inset to help elucidate subtle differences between the various salt concentrations.

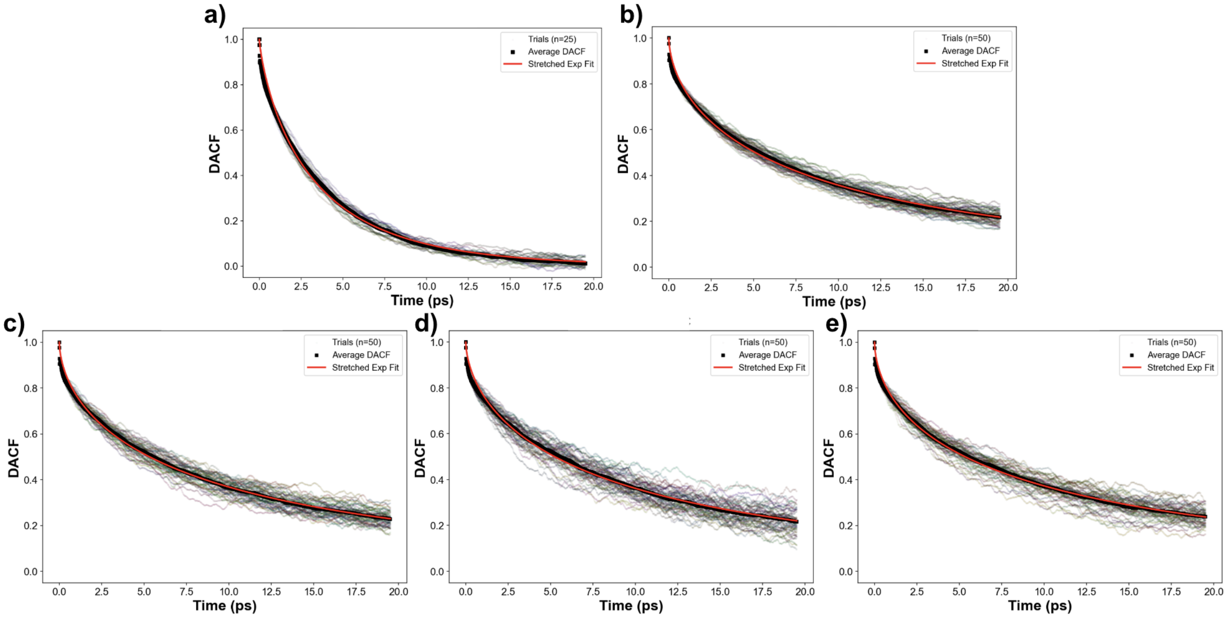

Figure S6: Normalized DACF data at 0 mM and 300K for bulk water (a) and hydration shell water for α-Syn (b), the N-Terminal domain (c), NAC domain (d), and C-Terminal domain (e). Each plot shows the every replicate simulation's data overlayed (n=25 for bulk, n=50 for hydration shell water), as well as an average of each trial with an accompanied stretched exponential fit for the averaged data points.

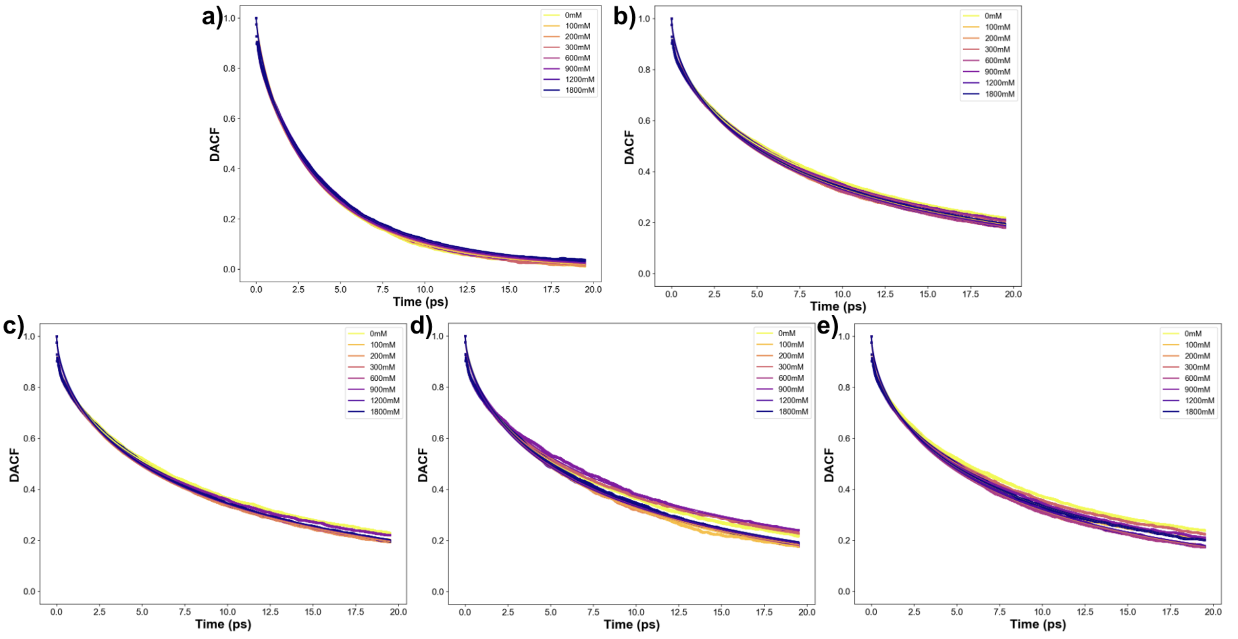

Figure S7: Averaged DACF data of each replicate simulation for each salt concentration tested at 300K for bulk water (a) and hydration shell water for α-Syn (b), the N-Terminal domain (c), NAC domain (d), and C-Terminal domain (e). Each set of averaged data is accompanied by a stretched exponential fit.

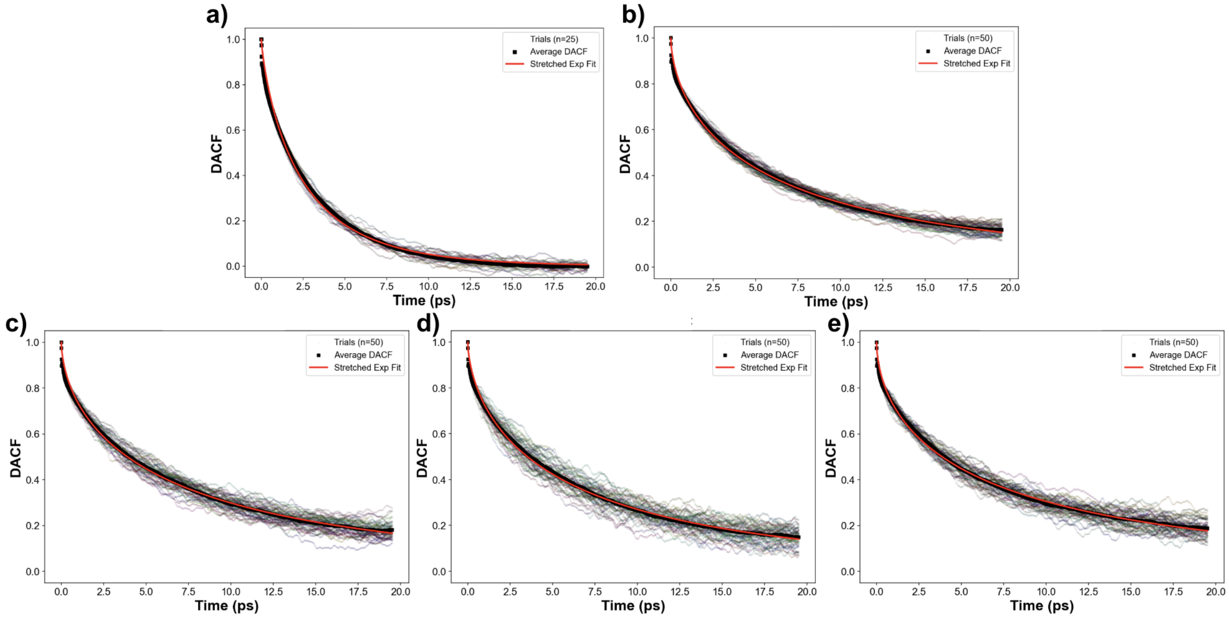

Figure S8: Normalized DACF data at 0 mM and 310K for bulk water (a) and hydration shell water for α-Syn (b), the N-Terminal domain (c), NAC domain (d), and C-Terminal domain (e). Each plot shows the every replicate simulation's data overlayed (n=25 for bulk, n=50 for hydration shell water), as well as an average of each trial with an accompanied stretched exponential fit for the averaged data points.

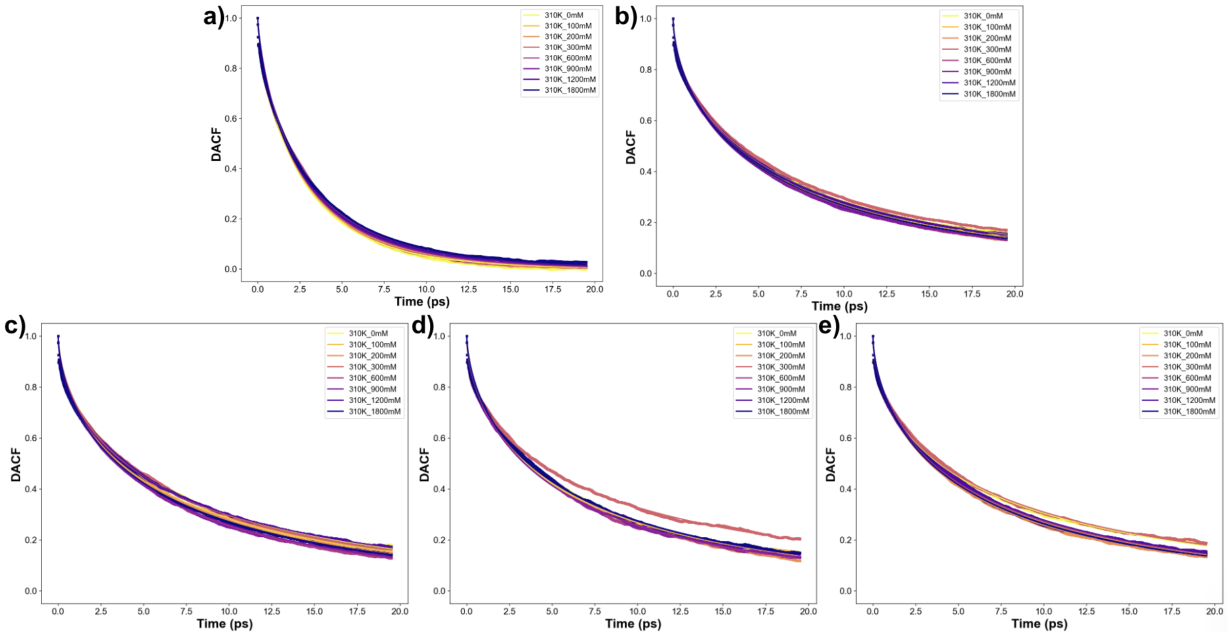

Figure S9: Averaged DACF data of each replicate simulation for each salt concentration tested at 310K for bulk water (a) and hydration shell water for α-Syn (b), the N-Terminal domain (c), NAC domain (d), and C-Terminal domain (e). Each set of averaged data is accompanied by a stretched exponential fit.

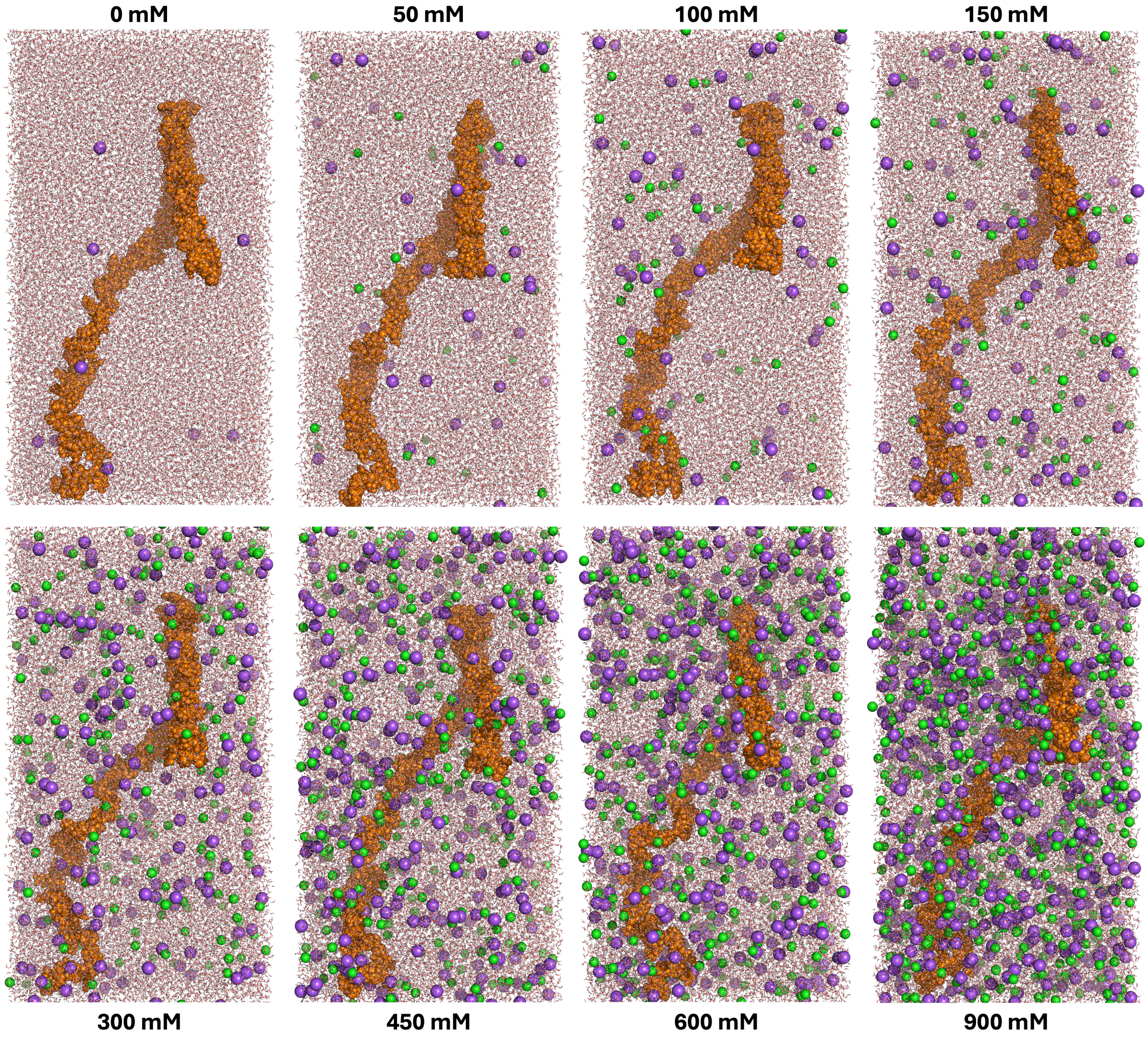

Figure S10: Snapshots highlighting the abundance of NaCl at the 8 different concentrations investigated.

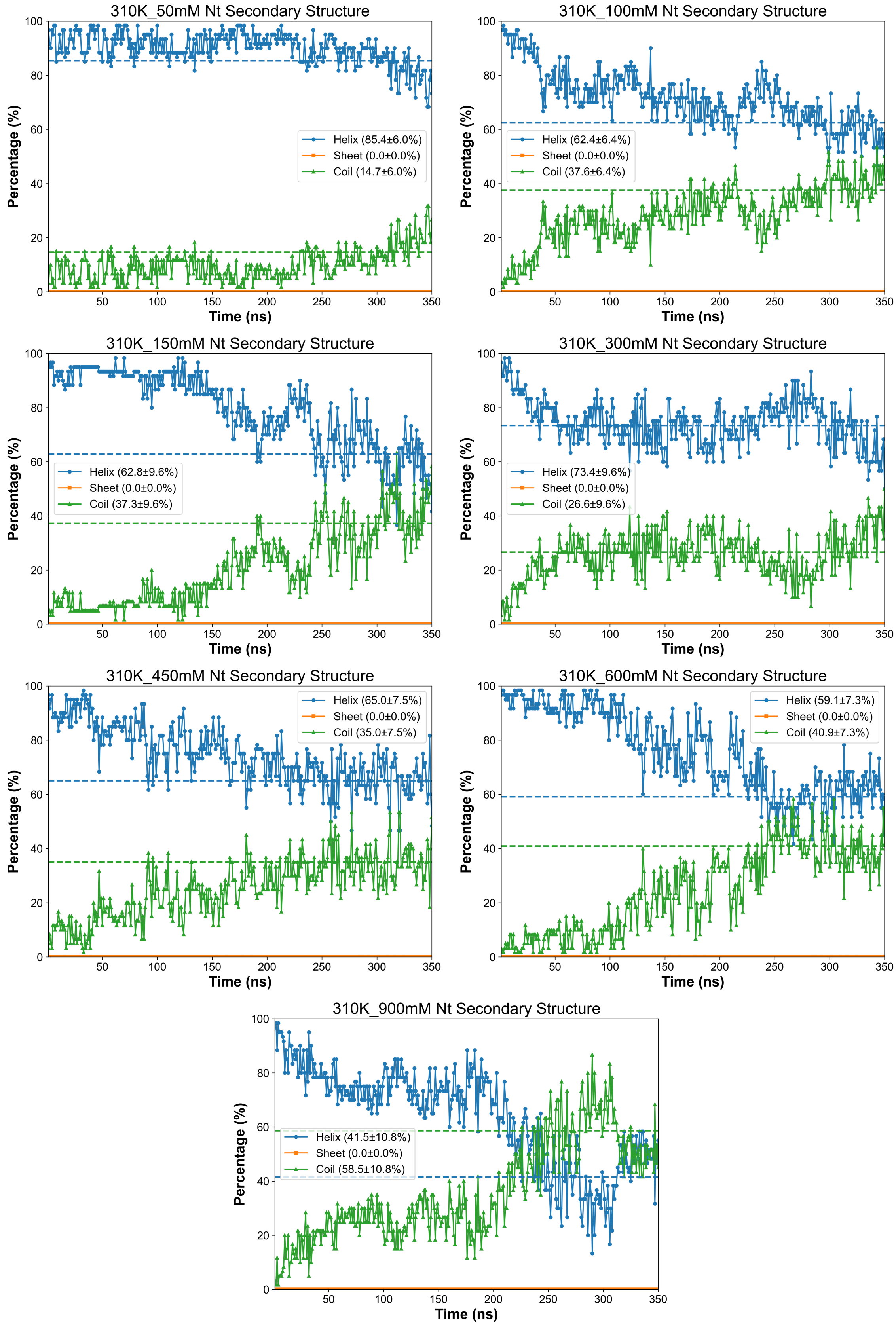

Figure S11: Secondary structure information of the N-terminal domain at 310 K.

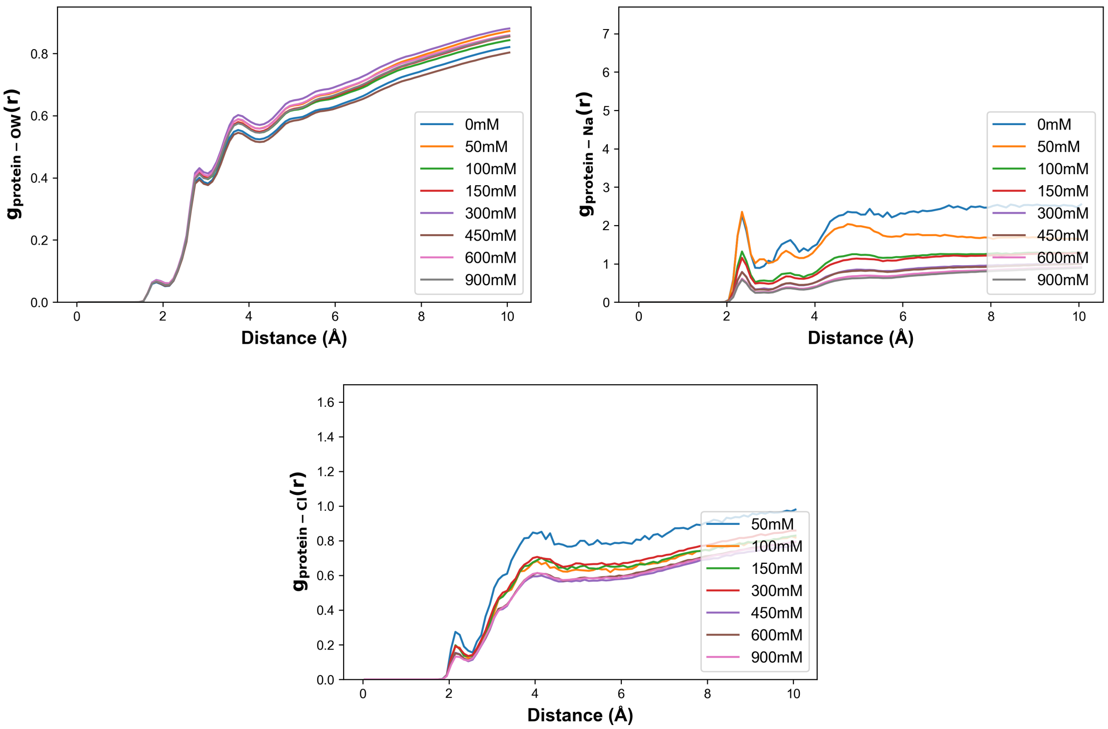

Figure S12: a) α-Syn-water oxygen radial pair distribution functions for the range of NaCl concentrations at 300 K. b) α-Syn-Na^{+} radial pair distribution functions for the range of NaCl concentrations at 300 K. c) α-Syn-Cl^{-} radial pair distribution functions for the range of NaCl concentrations at 300 K.

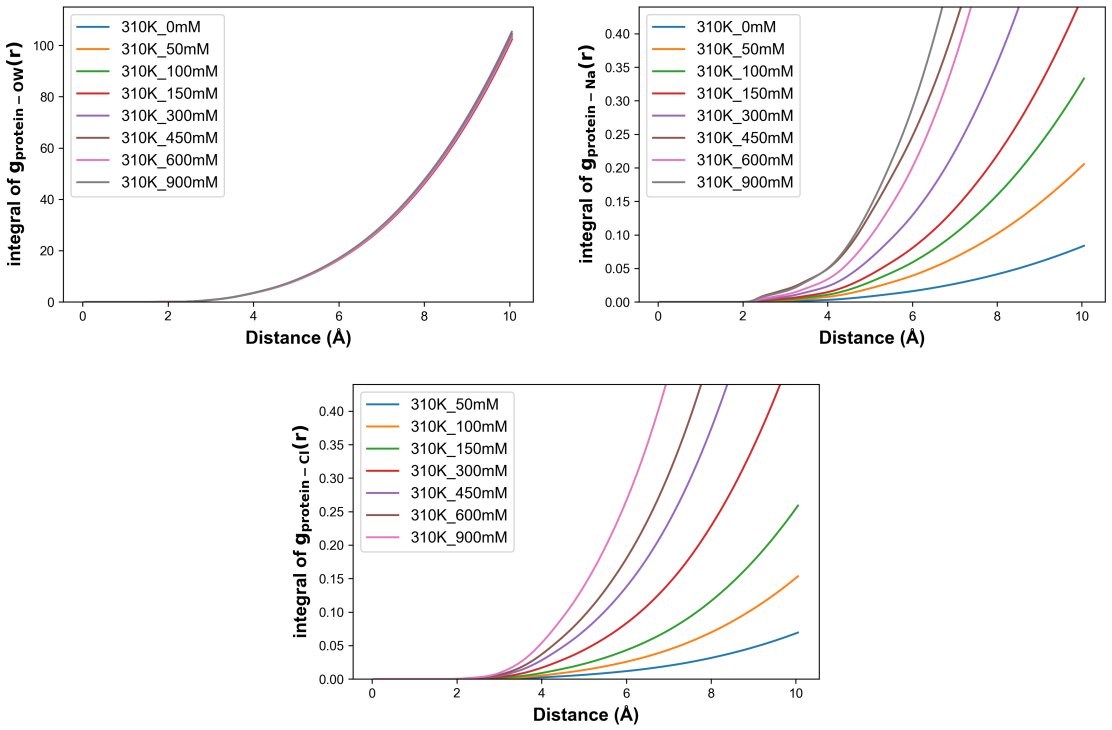

Figure S13: a) Coordination number for α-Syn-water for the range of NaCl concentrations at 310 K. b) Coordination number for α-Syn-Na^+ for the range of NaCl concentrations at 310 K. c) Coordination number for α-Syn-Cl^- for the range of NaCl concentrations at 310 K.

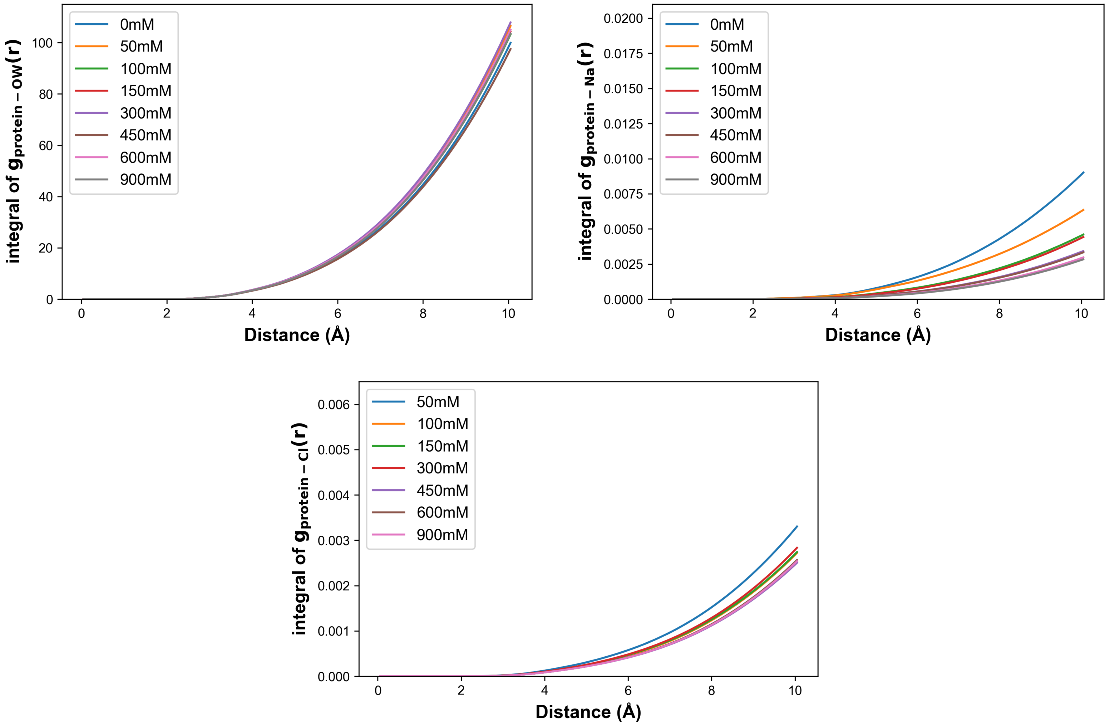

Figure S14: a) Coordination number for α-Syn-water for the range of NaCl concentrations at 300 K. b) Coordination number for α-Syn-Na^+ for the range of NaCl concentrations at 300 K. c) Coordination number for α-Syn-Cl^- for the range of NaCl concentrations at 300 K.

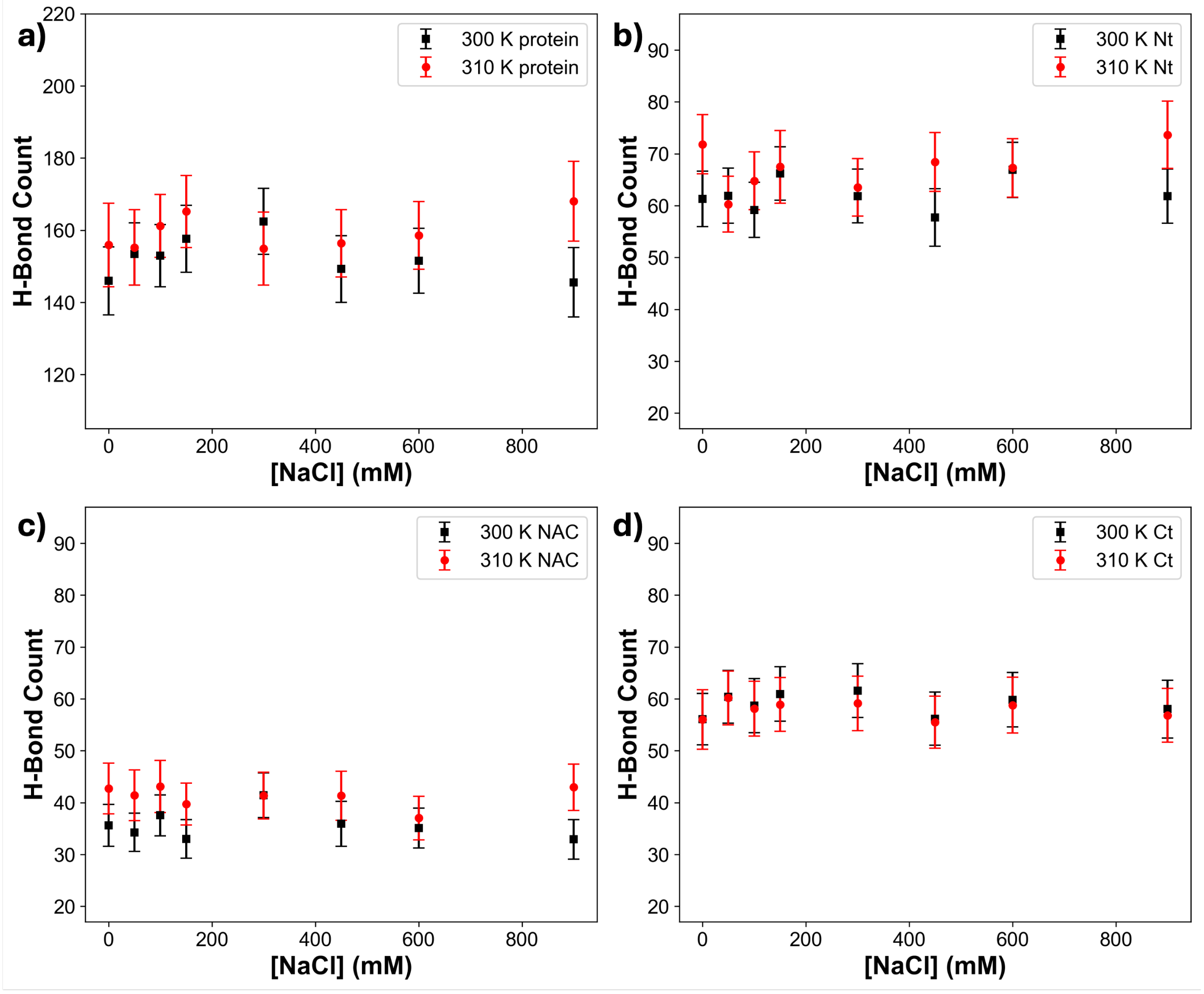

Figure S15: The average (and standard deviation) number of hydrogen bonds between α-Syn and water with 3.5 $\mathring{A}$ and 30°distance and angle cutoffs, respectively. This compares the systems at 300 K (black squares) and at 310 K (red circles) at each concentration of NaCl for the a) whole protein, b) N-terminal domain, c) NAC domain, and d) C-terminal domain.

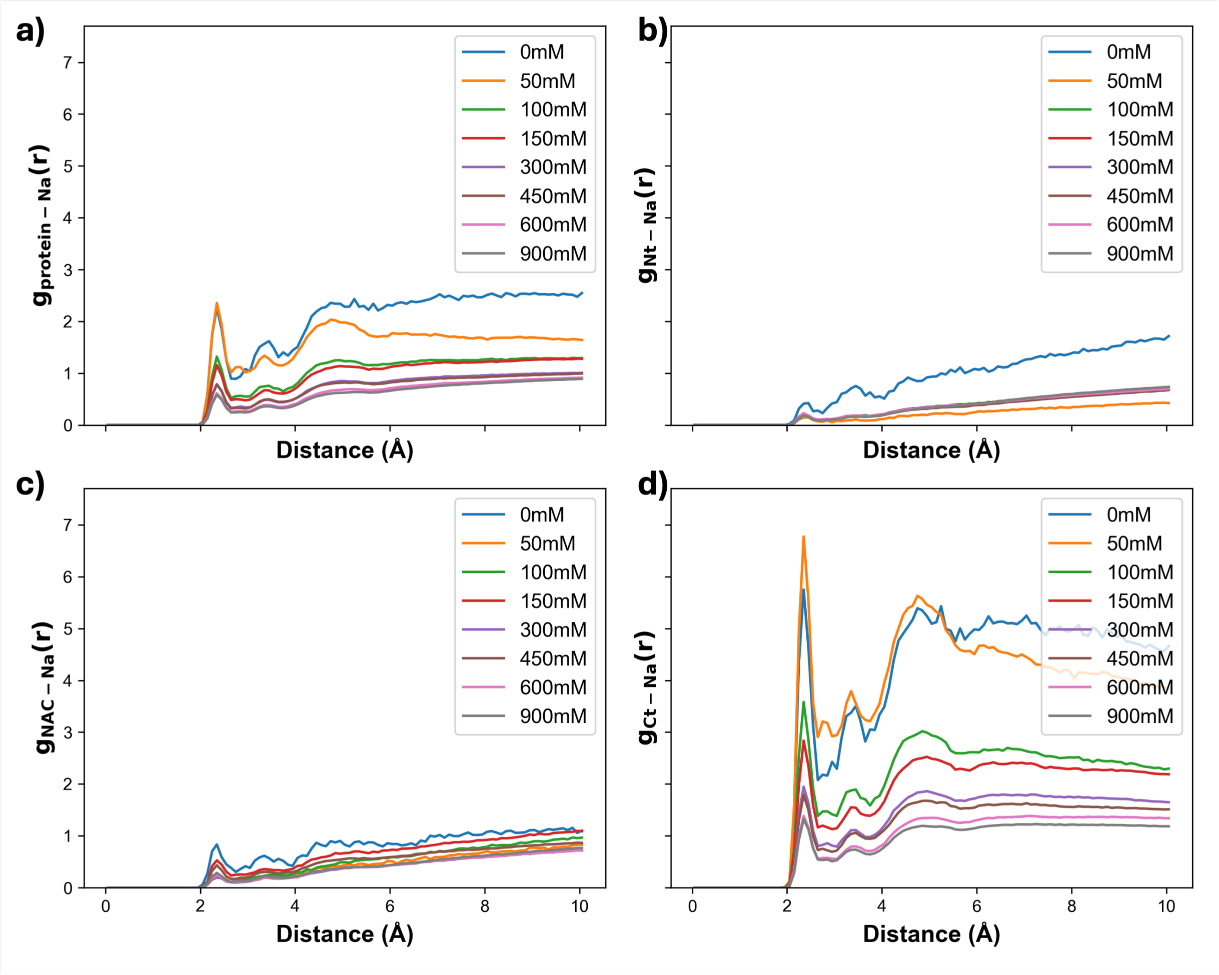

Figure S16: The α-Syn-Na^+ RDF at each concentration (at 300 K) for the a) whole protein, b) N-terminal domain, c) NAC domain and d) C-terminal domain.

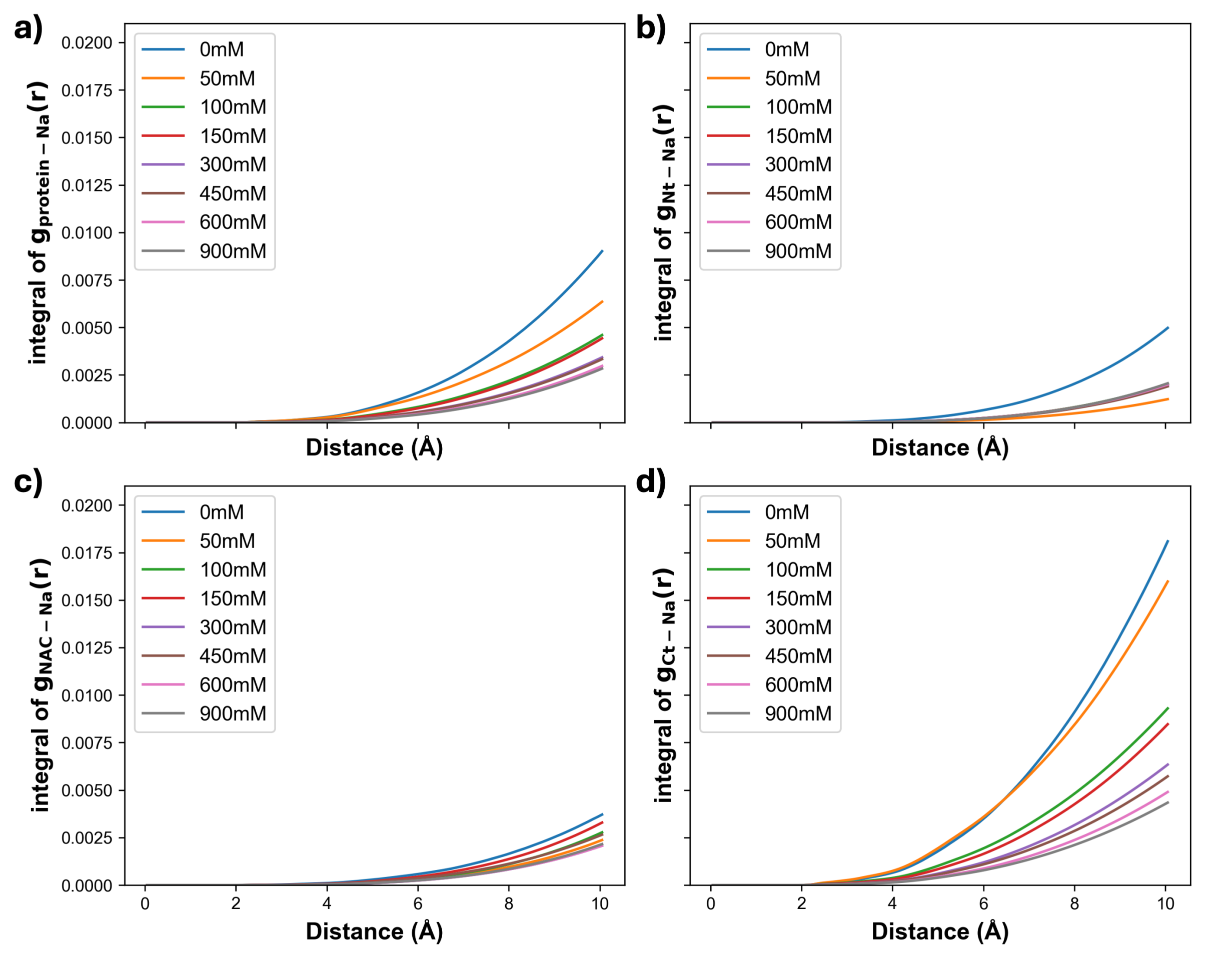

Figure S17: The integral for the α-Syn-Na^+ RDF at each concentration (at 300 K) for the a) whole protein, b) N-terminal domain, c) NAC domain and d) C-terminal domain.

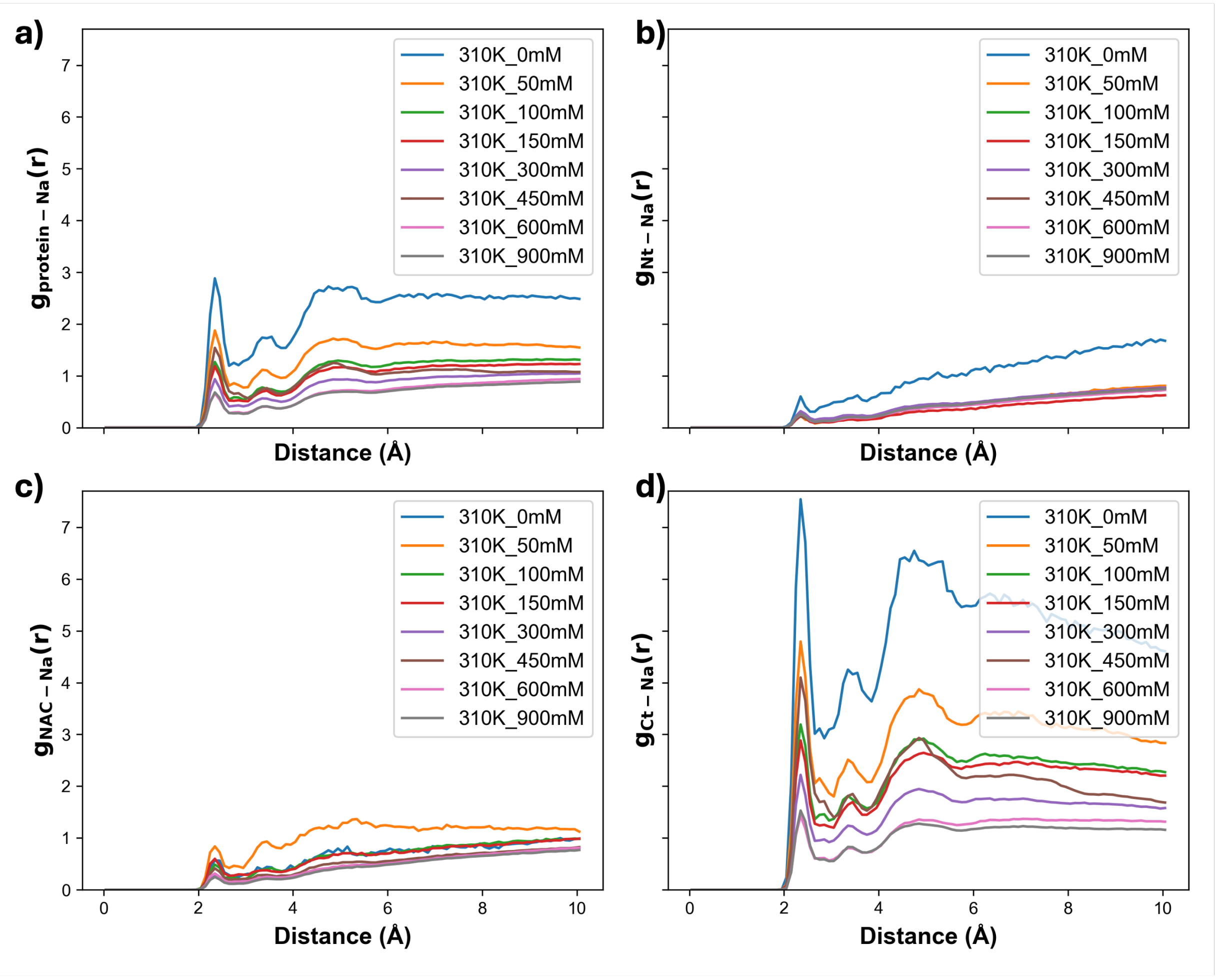

Figure S18: The α-Syn-Na^+ RDF at each concentration (at 310 K) for the a) whole protein, b) N-terminal domain, c) NAC domain and d) C-terminal domain.

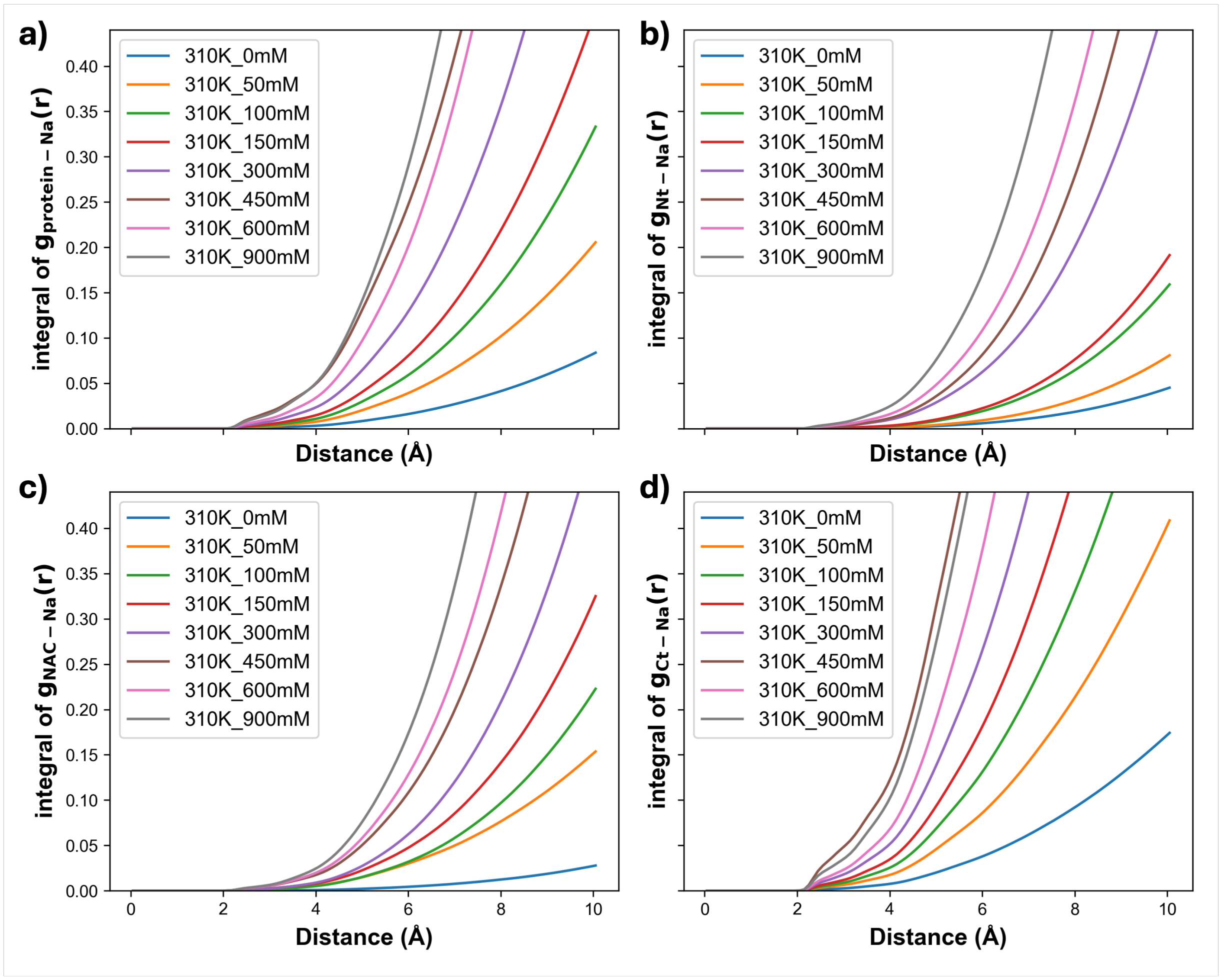

Figure S19: The integral for the α-Syn-Na^+ RDF at each concentration (at 310 K) for the a) whole protein, b) N-terminal domain, c) NAC domain and d) C-terminal domain.

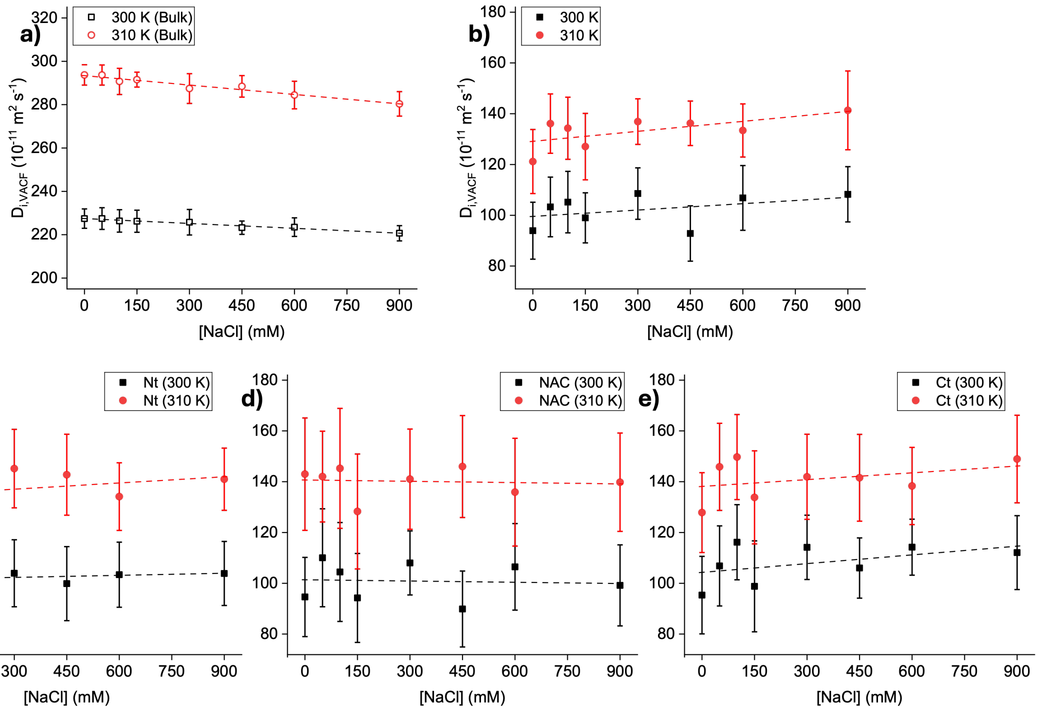

Figure S20: The calculated diffusion coefficient of water at various salt concentrations at both 300 K (black) and 310 K (red) in the bulk (a), as well as all of α-Syn (b) and the N-terminal, NAC, and C-terminal domains (c, d, and e, respectively).

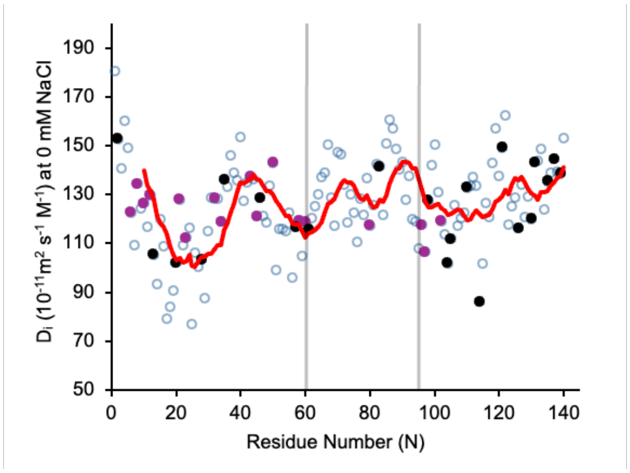

Figure S21: Diffusion coefficient (D_i), computed from VACF, of hydration water per residue at 310 K.
